# Executive Functions in Insomnia Disorder: A Systematic Review and Exploratory Meta-Analysis

**DOI:** 10.3389/fpsyg.2019.00101

**Published:** 2019-01-30

**Authors:** Andrea Ballesio, Maria Raisa Jessica V. Aquino, Simon D. Kyle, Fabio Ferlazzo, Caterina Lombardo

**Affiliations:** ^1^Department of Psychology, Faculty of Medicine and Psychology, Sapienza University of Rome, Rome, Italy; ^2^Primary Care Unit, Department of Public Health and Primary Care, University of Cambridge, Cambridge, United Kingdom; ^3^Sleep and Circadian Neuroscience Institute, Nuffield Department of Clinical Neurosciences, University of Oxford, Oxford, United Kingdom

**Keywords:** insomnia, executive functions, inhibition, working memory, flexibility, prefrontal cortex, cognition

## Abstract

**Background:** Executive functions (EFs) are involved in the control of basic psychological processes such as attention and memory and also contribute to emotion regulation. Research on the presence of EFs impairments in insomnia yielded inconsistent results. Therefore, we performed a systematic review of the literature on three EFs: inhibitory control, working memory, and cognitive flexibility in adults with insomnia in order to investigate the presence and magnitude of insomnia-related EFs impairments.

**Methods:** PubMed, Scopus, Medline, and PsycINFO were searched. Risk of bias assessment of included studies was performed by two independent researchers. Findings were summarised using both a narrative approach and meta-analysis. Cohen's *d* was calculated at 95% confidence interval (CI) as effect size of between groups differences.

**Results:** Twenty-eight studies comparing adult individuals with a diagnosis of insomnia and healthy controls on neuropsychological measures of EFs were included. Narrative synthesis revealed substantial variability across study findings. Factors that were primarily hypothesised to account for this variability are: objective sleep impairments and test sensitivity. Exploratory meta-analysis showed impaired performance of small to moderate magnitude in individuals with insomnia as compared to controls in reaction times, but not accuracy rates, of inhibitory control (*d* = −0.32, 95% CI: −0.52 to −0.13) and cognitive flexibility tasks (*d* = −0.30, 95% CI: −0.59 to −0.01). Performance in working memory tasks was also significantly impacted (*d* = −0.19, 95% CI: −0.38 to −0.00). Effects sizes were larger when insomnia was associated with objective sleep impairments, rather than normal sleep.

**Conclusions:** We gathered evidence supporting small to moderate deficits in EFs in individuals with insomnia. Due to the small sample size results should be considered preliminary and interpreted carefully.

## Introduction

### Rationale

Insomnia disorder is defined by difficulty falling asleep, maintaining sleep or early morning awakenings (American Psychiatric Association, 2013). Concomitant daytime consequences such as fatigue, reduction in motivation and energy, mood instability, and cognitive impairments are crucial components of insomnia (Shekleton et al., 2010). Symptoms should present at least three times a week over a period of 3 months to meet the diagnostic criteria for insomnia (American Psychiatric Association, 2013). Epidemiological data estimate the prevalence of insomnia disorder from 6 to 20% in industrialised societies, with rates varying depending on country under study and methodological quality (Calem et al., 2012; Chaput et al., 2018).

Deficits in the control of cognitive and emotional processes are key characteristics of insomnia, as highlighted by influential models on the disorder (Perlis et al., 1997; Harvey, 2002; Espie et al., 2006). For instance, according to Harvey (2002), patients with insomnia are unable to exert control over night-time intrusive cognitions and engage in diurnal repetitive and unwanted thoughts, such as ruminations, i.e., passively and repetitively focusing on the consequences of insomnia. Impairments in the domain of concentration, memory, attention, and emotion regulation are also generally reported in this population (Kyle et al., 2013; Harris et al., 2015; Cellini, 2016). Taken together, these findings raise the question of whether executive functions (EFs), the higher order cognitive processes which exert top-down control over basic psychological functions like attention, memory, and contribute to emotion regulation (Diamond, 2013; Yang et al., 2016), are also impacted in insomnia. To answer this question, we aimed to conduct a systematic review and exploratory meta-analysis of the literature examining EFs in insomnia disorder in an adult population. Before introducing our study, we discuss conceptual models of EFs, their clinical correlates and the role of EFs in insomnia.

EFs are considered top-down, higher-order cognitive processes needed to control and coordinate lower-level mental processes such as memory encoding and retrieval, orienting attention, and emotion regulation, which together enable self-regulation and contribute to goal-directed behaviour (Diamond, 2013; Snyder et al., 2015). Neuropsychological and neuroimaging studies in clinical and healthy populations suggest that the prefrontal cortex (PFC) as well as parietal and cerebellar networks subserve EFs (Nowrangi et al., 2014; Yuan and Raz, 2014). However, the definition and conceptualisation of EFs remains inconsistent in the literature. Indeed, there is a lack of agreement regarding whether EFs should be considered unitarily (e.g., Duncan, 2010) or as a number of different and independent cognitive processes (e.g., Miyake et al., 2000). Moreover, conceptualisation and definition of EFs varies substantially depending on the field of study and the population of interest. This is reflected by the prevalent use of the term EFs in neuropsychology (aimed at the assessment of patients) and the term control processes in the cognitive sciences (typically aimed at investigating in healthy populations the cognitive mechanisms underlying the EFs). Given the clinical nature of this review, we use the terms EFs throughout the text.

Different conceptualisations and classifications of EFs have been developed over several decades (see Gratton et al., 2017 for a review). Recently, an influential model hypothesised that the performance on complex EF tasks is underpinned by three core EFs: inhibitory control, working memory, and cognitive flexibility (Miyake et al., 2000; Diamond, 2013). Inhibitory control refers to the ability to reduce the effect of strong internal predispositions, automatic schemata or responses when they are not useful for accomplishing the task goal. Inhibitory control is therefore needed to suppress thoughts, emotions, motor responses and irrelevant stimuli (Aron et al., 2004). Working memory involves the ability to hold and manipulate goal-related information in mind (Repovs and Baddeley, 2006). Finally, cognitive flexibility refers to the ability to readily change perspective, demands or priorities, and to quickly adjust from set-shifting (Miyake et al., 2000). Given its wide use in cognitive and clinical literature, we decided to focus the present systematic review on the tripartite model of EFs, based on inhibitory control, working memory, and cognitive flexibility. This tripartite model of EFs also overlaps with the model of “executive control” adopted in insomnia research by Vgontzas et al. (2013) in their attempt to identify different phenotypes of insomnia from symptom severity and biological correlates, as further described below.

Although individuals with insomnia commonly report subjective difficulties in different cognitive functions involving executive control like attention, memory, and concentration (Kyle et al., 2013; Harris et al., 2015; Cellini, 2016), objective EFs deficits have been difficult to capture through standardised measures in laboratory settings. Additionally, few reviews on EFs in insomnia have been published to date. A review on daytime impairments in insomnia concluded that on tests of attentional shifting and working memory, individuals with insomnia generally perform worse than good sleepers (Shekleton et al., 2010). However, the authors did not include these tests within the EFs domain and instead included planning, reasoning, flexibility, and multitasking in this category. Performance on these EFs were mostly preserved in individuals with insomnia.

Recent evidence suggests relevant clinical correlates of EFs impairments that may be of particular interest for insomnia research. For instance, EFs have been associated with poor cognitive self-regulatory strategies, including rumination. A recent meta-analysis of correlational studies showed that poorer inhibitory control and cognitive flexibility were significantly associated with higher rumination in the general population (Yang et al., 2016). This finding may be particularly relevant for insomnia, as most theoretical models suggest a role of repetitive negative thinking such as rumination and the implementation of thought control strategies in the maintenance of the disorder (e.g., Harvey, 2002). In line with this, we recently found that rumination about symptoms of insomnia was associated with poor EFs in a clinical sample (Ballesio et al., 2018). Additionally, poor EFs have been associated with significant impairment in instrumental activities of daily living (Vaughan and Giovanello, 2010), which may contribute to lower quality of life in insomnia patients (Kyle et al., 2013) and increase indirect costs associated with insomnia (e.g., due to errors in workplace; Gustavsson et al., 2011). These potential clinical correlates of EFs in insomnia are therefore further grounds to systematically review the literature on EFs in this population.

To date, only one meta-analysis has investigated executive performance in insomnia (Fortier-Brochu et al., 2012). This included cross-sectional studies investigating daytime cognitive performance in adults with insomnia and good sleepers and published up to 2009. Findings showed that individuals with insomnia perform significantly worse than controls on tasks measuring manipulation and retention of information in working memory, with effect sizes of medium magnitude (*d* = 0.42). Small and non-significant effects were found with respect to tasks assessing inhibitory control (*d* = 0.19) and cognitive flexibility (*d* = 0.16). However, 6 years have passed since Fortier-Brochu et al.'s (2012) systematic review and meta-analysis on cognitive functions in insomnia, raising the need to appraise and summarise the state of the evidence again.

Among the factors that were previously hypothesised to account for variability between studies' findings, objective sleep received particular attention. In a large population-based study, Fernandez-Mendoza et al. (2010) concluded that only individuals with insomnia with objective short sleep duration, measured through polysomnographic records, showed impairments in executive control tasks. A subsequent theoretical review suggested that impairments in higher order cognitive processes in insomnia may be present only when the disorder is associated with shortened sleep duration (Vgontzas et al., 2013). This hypothesis may partly explain the inconsistency found in previous research on EFs. Nevertheless, it has never been tested in a systematic search of the literature. Other factors have been hypothesised to account for variability in previous results. For instance, it has been suggested that individuals with insomnia may engage increased cognitive effort in high cognitive load tasks to compensate for their deficits (Schmidt et al., 2014). Moreover, “time of the day” has been considered a confounding factor, since it is possible that individuals with insomnia and good sleeper controls have different underlying circadian rhythms, and by extension differentially affecting patterns of cognitive performance (see Shekleton et al., 2010 for a review).

### Objective

To conduct a systematic review of the literature on inhibitory control, working memory, and cognitive flexibility in individuals with insomnia.

### Research Questions

1. Are inhibitory controls, working memory, and cognitive flexibility impacted in individuals with a diagnosis of insomnia disorder?
2. Is there a relationship between objective sleep and EFs deficits in insomnia disorder?

## Methods

### Study Design

This study was conducted according to the preferred reporting items for systematic review and meta-analysis (PRISMA) guidelines (Moher et al., 2009) (see the PRISMA Checklist reported in Document S1).

### Participants, Interventions, Comparators

The following inclusion criteria were applied to identified records: (1) presence of a group of adult individuals with clinical insomnia, (2) presence of a control group, (3) presence of at least one neuropsychological test assessing inhibitory control and/or working memory and/or cognitive flexibility. Given that EFs may be affected by psychoactive substances (Killgore et al., 2009, 2014), studies which allowed participants to take psychoactive medication, as well as caffeine and/or alcohol were excluded. Moreover, given that comorbid disorders may similarly affect EFs (Snyder et al., 2015), studies conducted on insomnia-comorbid samples were excluded. Studies dealing with sleep-related attentional bias were not included, as they have been recently systematically reviewed elsewhere (Harris et al., 2015). Additionally, only studies providing data to compute effect sizes were included in the meta-analytic calculations.

### Search Strategy

The literature search was performed by the first author using two strategies. First, PubMed, Scopus, Medline, and PsycINFO were searched from inception to 10th August 2018 using the following keywords: “insomnia” or “sleep disturbance” and “executive function^*^” or “inhibition” or “inhibitory control” or “working memory” or “flexibility.” Second, the reference lists of relevant review articles were searched. When titles of studies appeared relevant to the present review, abstracts and full-texts were screened against the eligibility criteria by the first author.

### Data Sources, Studies Sections, and Data Extraction

For the qualitative synthesis, data on a number of procedural variables were extracted by the first author from included studies including demographic and clinical characteristics of the sample, as well as methodological variables. Given the importance of objective sleep duration which is hypothesised to contribute to executive dysfunction (Fernandez-Mendoza et al., 2010; Vgontzas et al., 2013), information about group differences between individuals with insomnia and controls on total sleep time (TST) based on polysomnographic or actigraphic recordings were extracted, together with information about objective sleep efficiency (SE). TST is generally calculated as time spent in bed during the night (total bed time, TBT) minus the time needed to fall asleep, the wake after sleep onset and early morning awakenings. SE is then calculated as TST/TBT^*^100 (Carney et al., 2012). For the meta-analysis, means and standard deviations of the indices of performance reported in the included studies were extracted by the first author to compute effect sizes. When means and standard deviations were not reported in the studies, effect sizes were computed from means and standard errors. Effect sizes were not estimated from graphs. To identify and categorise the neuropsychological tests, we referred to recent systematic reviews on the topic (Diamond, 2013; Snyder et al., 2015) and consulted systematic reviews and meta-analyses on cognitive impairment in insomnia (Shekleton et al., 2010; Fortier-Brochu et al., 2012). Due to variations in test categorisation in the domain of EFs, we decided to follow the categorisation used in the meta-analysis of Fortier-Brochu et al. (2012), drawn by two independent neuropsychologists.

### Data Analysis

Two independent investigators (AB, RA) assessed risk of bias using the checklist for assessing the quality of quantitative studies (Kmet et al., 2004). This tool appraises studies on different potential areas of bias, including appropriateness of the design, method of subject selection, blinding procedure, sample size, and data analysis. Disagreements between the investigators was resolved by consensus discussion.

Given the high variability of EFs measures, findings were first summarised and discussed using narrative synthesis (Popay et al., 2006). This allowed for the discussion of the differences in study findings and how clinical and methodological variables might have influenced study results, including between group differences on objective sleep.

When variability in EFs measures was limited, and there was a relevant number of studies to analyse (at least 3), meta-analysis was used in addition to the narrative synthesis to statistically estimate the presence and magnitude of EFs impairments. This was possible for the EFs assessed through reasonably comparable tasks (i.e., similar paradigms and outcomes) and for studies providing data to calculate effect sizes. To limit the impact of outcome measures' variability, analyses were run separately according to outcome type, i.e., reaction times and accuracy. When there was a relevant number of studies to analyse (at least 3), we ran sensitivity analysis to investigate the impact of objective sleep impairments on EFs.

For the meta-analytic calculations, standardised mean differences (Cohen's *d*) were estimated at 95% confidence intervals for group differences on cognitive tasks performance. Cohen's *d* was derived by subtracting the mean for control groups from the mean for insomnia groups and dividing the result by the pooled standard deviation. The direction of effect size values was adjusted so that negative effects always indicate poorer performance in individuals with insomnia compared to controls. Meta-analytic calculations were computed using the statistical software “Comprehensive Meta-Analysis” version 2. A fixed effects model was used following the procedure of other authors (Fortier-Brochu et al., 2012). To test heterogeneity of effects distribution (i.e., variability in the distribution of effect sizes across studies included in a meta-analysis), Cochran's *Q* and Higgins's *I*^2^ were calculated. Cochran's *Q* is computed as a weighted sum of squared differences between single study effects and the pooled effect across studies. Significant values indicate high level of heterogeneity between effects that need to be further investigated. Higgins's *I*^2^ assesses the variability in effect estimates that is due to between-study heterogeneity rather than to chance. Low percentages of *I*^2^ are indicative of low heterogeneity while percentages over 75% represent considerable levels of heterogeneity.

Additionally, we performed a series of subgroup analyses including either studies that reported significant sleep differences (objective TST or SE) between those with insomnia and good sleepers or studies reporting comparable sleep values between groups in order to investigate the differences in effect sizes between these sets of studies. This allowed us to investigate the effects of insomnia with objective sleep impairment vs. insomnia with normal sleep on EFs.

## Results

### Study Selection and Characteristics

**Figure 1:**
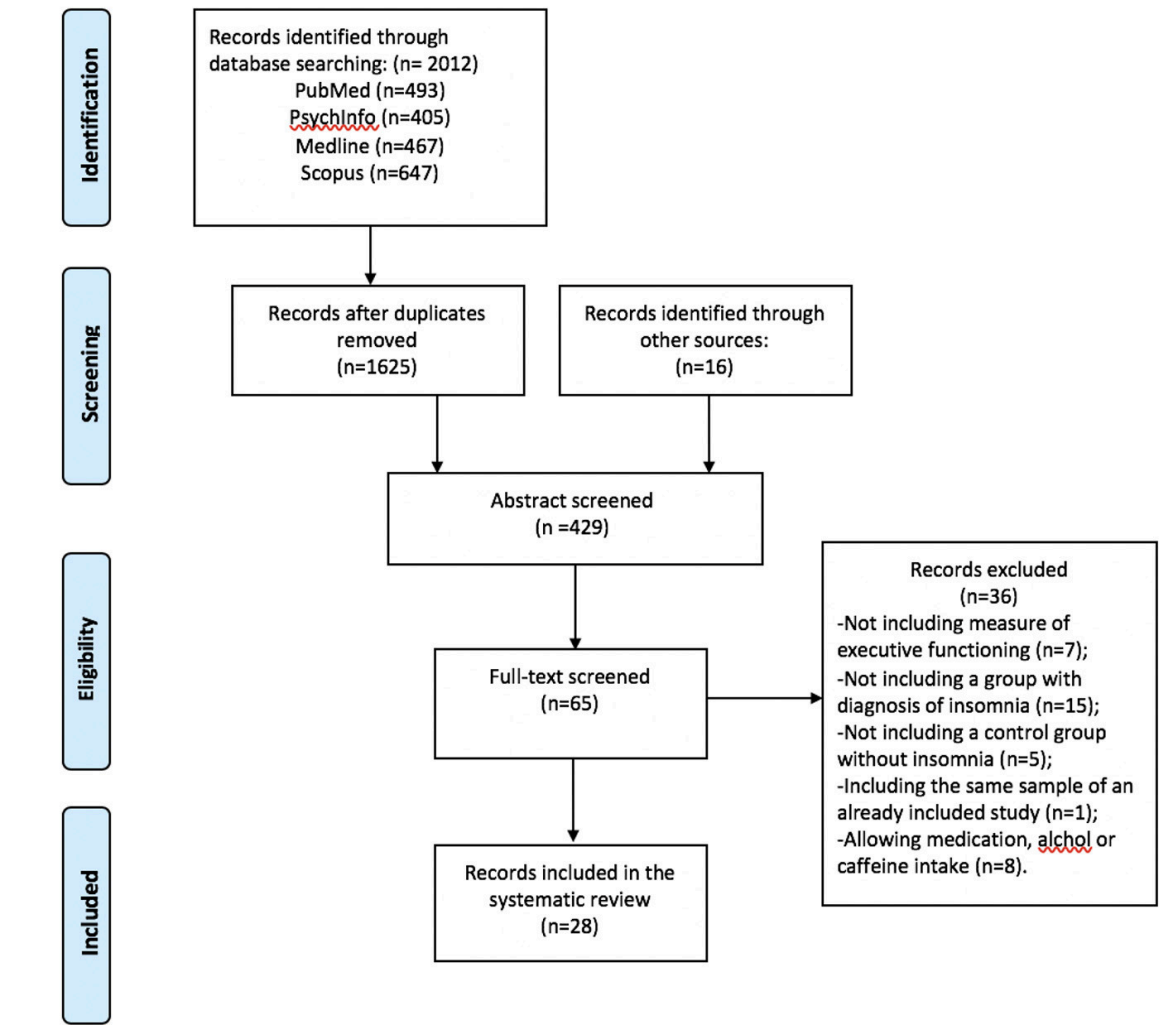
Flow chart for study selection.

The study selection flowchart is reported in Figure 1. A detailed description of study characteristics is provided in Table Table 1. Database search yielded 2012 studies (PubMed = 493, PsycINFO = 405, Medline = 467, Scopus *n* = 647). After removing duplicates, 1,625 records were identified. Reference screening yielded 16 additional records. In sum, 429 abstracts and 65 full-texts were screened against the eligibility criteria. Thirty-six studies were excluded because of the absence of: measures of inhibitory control, working memory or cognitive flexibility (*n* = 7), a group of individuals with standardized diagnosis of insomnia disorder (*n* = 15), and a control group (*n* = 5). A further eight studies were excluded as these allowed participants to take drugs or psychoactive substances (e.g., caffeine or alcohol) prior to assessment. Furthermore, one study was excluded due to it being a secondary analysis of a study already included for review (see Document S1 for excluded studies). Finally, 28 studies met the inclusion criteria and were included in the systematic review.

**Table 1:** Study characteristics.

**Author (year)**	**Diagnostic criteria**	**Insomnia duration**	**Assessment**	**Comorbidity**	***N* insomnia**	***N* controls**	**%Female insomnia**	**% Female controls**	**Age insomnia (M)**	**Age controls (M)**	**Pharmaco intake**	**Caffeine intake**	**Alcol intake**	**Time of testing**
Altena et al., 2008	RDC, Lichstein criteria	>2.5 years	Questionnaire	Subclinical psychiatric symptoms	21	12	80.9	75	61	60	Excluded	n/a	n/a	PM
Backhaus et al., 2006	DMS-IV	9.2 years	Clinical interview	Excluded	15	13	50	61.3	41.6	40.1	Excluded	n/a	n/a	AM
Bonnet and Arand, 1995	SOL>45 or WASO>60 min, 4 nights per week for 1 year	8 years	Questionnaire	Excluded	10	10	n/a	n/a	38.3	38.6	Excluded	Excluded	n/a	Repeated testing
Cellini et al., 2014	RDC	n/a	Clinical interview, questionnaire	Excluded	13	13	61.5	46.1	23.3	24.3	Excluded	Excluded	Excluded	PM
Chen et al., 2016	ICSD-3	>6 months	Questionnaire	Excluded	21	20	71.4	65	41.8	38.1	n/a	n/a	n/a	AM
Covassin et al., 2011	DSM-IV	n/a	Clinical interview, questionnaire	Excluded	8	8	50	50	22.9	24.8	Excluded	Excluded	Excluded	Repeated testing
Crenshaw and Edinger, 1999	DMS-III-R	n/a	Clinical interview	Excluded	32	32	50	50	67.7	67.5	Excluded	n/a	n/a	Repeated testing
Edinger et al., 2000	DMS-III-R	>6 months	Clinical interview	Excluded	27	31	44.4	51.6	49.7	46.4	Excluded	n/a	n/a	Repeated testing
Edinger et al., 2008	DSM-IV	n/a	Clinical interview	Excluded	79	84	54.4	48.8	50	48.6	Excluded	n/a	n/a	Repeated testing
Fang et al., 2008	DSM-IV	n/a	Questionnaire, actigraphy	Excluded	18	21	66.7	66.7	34.1	27.8	n/a	Excluded	Excluded	PM
Fortier-Brochu and Morin, 2014	DSM-IV, ICD-10	17.3 years	Clinical interview, diary	Excluded	25	16	56	50	44.4	42.8	Excluded	Excluded	Excluded	AM
Guo et al., 2017	DSM-IV	n/a	Questionnaire	Excluded	40	48	77.5	72.9	37.3	39.8	Excluded**	n/a	n/a	n/a
Haimov et al., 2008	Lichstein criteria	n/a	Questionnaire, actigraphy	Excluded	35	64	57.1	67.2	73.6	71.6	Excluded	n/a	n/a	PM
Joo et al., 2013	ICSD-2	>1 year	Clinical interview, diary, questionnaire	Excluded	27	27	92.5	85	52.3	51.7	Excluded	Excluded	Excluded	n/a
Khassawneh et al., 2018	DSM-5, ICSD-3	n/a	Clinical interview, questionnaire, psg	Excluded	35	54	71.4	70.4	40.6	31.5	Excluded	n/a	n/a	Repeated testing
Liu et al., 2014	DSM-IV	6.4 years	Clinical interview, questionnaire	Excluded	36	26	58.3	61.5	42.9	40.5	Excluded	Excluded	Excluded	n/a
Lovato et al., 2013	DSM-IV	n/a	Clinical interview, questionnaire	Excluded	49	49	55.2	55.2	69.4	70	Excluded	n/a	n/a	n/a
Noh et al., 2012	ICSD-2	7.6 years	Clinical interview	Excluded	20	20	90	90	50.8	50.4	Excluded	Excluded	Excluded	n/a
Perrier et al., 2015	DSM-IV	n/a	Clinical interview, questionnaire	Excluded	21	16	57.1	62.5	48.7	48.3	Excluded	n/a	n/a	PM
Randazzo et al., 2000	DSM-IV	n/a	n/a	Excluded	35	35	71.4	77.1	43.6	43.5	n/a	n/a	n/a	n/a
Rosa and Bonnet, 2000	SOL>45 or WASO>60 min, 4 nights per week for 1 year	7.9 years	Questionnaire, psg	Excluded	121	56	38	32	35	36	Excluded	Excluded	n/a	Repeated testing
Sagaspe et al., 2007	ICSD-2	>6 months	Clinical interview, psg	n/a	13	13	38.4	38.4	47.4	47.4	Excluded	n/a	n/a	PM
Son et al., 2018	DSM-5	4.9 years	Clinical interview, questionnaire	Excluded	21	26	57.1	57.7	36.6	33.2	Excluded	n/a	n/a	AM
Shekleton et al., 2014	RDC	n/a	Clinical interview, questionnaire	Excluded	76	20	57.8	70	35.7	34.7	Excluded	Excluded	n/a	Repeated testing
Siversten et al., 2013	DSM-IV	n/a	Questionnaire	Excluded	30	91	n/a	n/a	n/a	n/a	n/a	n/a	n/a	n/a
Szelenberger and Niemcewicz, 2001	DSM-IV	8.2 years	Questionnaire, actigraphy, psg	Excluded	14	14	57.1	57.1	41.2	36.2	Excluded	n/a	n/a	AM
Varkevisser et al., 2007	ICSD-2	n/a	Clinical interview, diary	n/a	39	20	58.9	50	40.9	42.6	n/a	n/a	n/a	Repeated testing
Vignola et al., 2000	ICSD, DSM-IV	n/a	Clinical Interview, questionnaire, diary	Excluded	20	20	10	10	61.7	63.3	Excluded benzodiazepine	n/a	n/a	AM

*AM, morning; DSM, diagnostic and statistical manual of mental disorders; ICD, international classification of diseases; ICSD, international classification of sleep disorders; n/a, not available; PM, afternoon/evening; PSG, polysomnography; RDC, research diagnostic criteria; SEI, sleep efficiency index; SOL, sleep onset latency; TST, total sleep time, WASO, wake after sleep onset. X = no group differences*,

** *excluded if could impact cognitive function or reaction times*.

Data from 901 participants with insomnia and 859 controls were qualitatively evaluated. Mean percentage of females was 58.9 in the insomnia group and 58.5 in the controls. Mean age was 45.6 years in the insomnia group and 44.4 years in the controls.

### Synthesized Findings

#### Narrative Synthesis

##### Inhibitory control

Thirteen studies reported a neuropsychological measure of inhibitory control as an outcome (Crenshaw and Edinger, 1999; Edinger et al., 2000, 2008; Szelenberger and Niemcewicz, 2001; Backhaus et al., 2006; Sagaspe et al., 2007; Haimov et al., 2008; Covassin et al., 2011; Joo et al., 2013; Siversten et al., 2013; Fortier-Brochu and Morin, 2014; Liu et al., 2014; Perrier et al., 2015). Three of these measured inhibitory control through the continuous performance test (Crenshaw and Edinger, 1999; Edinger et al., 2000, 2008) and one through the continuous performance test II (Fortier-Brochu and Morin, 2014). Two studies used the Stroop test (Haimov et al., 2008; Joo et al., 2013), one a similar colour-word interference test (Siversten et al., 2013) and two the stop-signal task (Sagaspe et al., 2007; Covassin et al., 2011). The attention network test, which evaluates three attention networks (alerting, orienting, executive control) was used by two studies (Liu et al., 2014; Perrier et al., 2015). Finally, one study used the go/no-go paradigm (Backhaus et al., 2006).

Six of thirteen studies reported significant differences between individuals with insomnia and controls on inhibitory performance and objective sleep (see below, Haimov et al., 2008; Covassin et al., 2011; Joo et al., 2013; Fortier-Brochu and Morin, 2014; Liu et al., 2014; Perrier et al., 2015).

Covassin et al. (2011) found that young adults with insomnia showed longer reaction times at the stop trials of the stop-signal task indicating poorer inhibitory control. However, in terms of accuracy, no differences were found. In contrast, Fortier-Brochu and Morin (2014) found that participants with insomnia differed from good sleepers in the number of perseverative errors of the continuous performance task II, but not on mean reaction times. Haimov et al. (2008) reported that individuals with insomnia showed longer reaction times in the Stroop test, consistent with Joo et al. (2013). Liu et al. (2014) found that adults with insomnia showed impaired functioning in the executive control performance of the attentional network test. In contrast, Perrier et al. (2015) found intact performance on the same task, but increased reaction times (RTs) in the incongruent Flankers compared to congruent and neutral Flankers, that they interpreted as a conflict resolution deficit. With respect to objective sleep, four of these studies reported significant shorter TST (Covassin et al., 2011; Joo et al., 2013; Fortier-Brochu and Morin, 2014; Liu et al., 2014) and two lower SE in insomnia as compared to controls (Haimov et al., 2008; Perrier et al., 2015).

Three of the eight studies that found no significant differences between individuals with insomnia and controls on inhibitory control tasks found no differences between groups on TST (Crenshaw and Edinger, 1999; Edinger et al., 2000, 2008), one in both TST and SE (Crenshaw and Edinger, 1999); three did not report information on objective sleep data (Szelenberger and Niemcewicz, 2001; Sagaspe et al., 2007; Siversten et al., 2013). Only in the study of Backhaus et al. (2006), participants with insomnia objectively slept less and worse than controls, although no effects on inhibitory control were found. Findings on inhibitory control are summarised in Table Table 2.

**Table 2:** Comparison of individuals with insomnia and controls on tasks of inhibitory control.

**Author (year)**	***n* Insomnia; controls**	**Task**	**Outcome**	**Group difference**	**Group differences TST**	**Group difference SEI**
Backhaus et al., 2006	15; 13	Go/no-go	Response times	X	*p* < 0.05	*p* < 0.05
			Number of correct responses	X		
Covassin et al., 2011	8; 8	Stop-Signal task	Go reaction times	X	*p* < 0.05	*p* < 0.05
			Stop reaction times	*p* < 0.05		
			Accuracy	X		
			Errors	X		
Crenshaw and Edinger, 1999	32; 32	Continuous performance task	Response latency	X	X	X
Edinger et al., 2000	27; 31	Continuous performance task	Response latency	X	X	*p* < 0.05
Edinger et al., 2008	79; 84	Continuous performance task	Response latency	X	X	*p* < 0.05
Fortier-Brochu and Morin, 2014	25; 16	Continuous performance task II	Perseverative errors	*p* < 0.05	*p* < 0.05	*p* < 0.05
Haimov et al., 2008	35; 64	Stroop	Response times	*p* < 0.05	n/a	*p* < 0.05
Joo et al., 2013	27; 27	Stroop	Response times	*p* < 0.05	*p* < 0.05	*p* < 0.05
Liu et al., 2014	36; 26	Attention network test	Response times (executive control)	*p* < 0.05	*p* < 0.05	*p* < 0.05
Perrier et al., 2015	21; 16	Attention network test	Response times (executive control)	X	X	*p* < 0.05
			Response times*	*p* < 0.05		
Sagaspe et al., 2007	13; 13	Stop-signal task	Go reaction times	X	n/a	n/a
			Stop reaction times	X		
			Accuracy	X		
			Errors	X		
Siversten et al., 2013	30; 91	Color-word interference task	Response times	X	n/a	n/a
Szelenberger and Niemcewicz, 2001	14; 14	Continuous attention task	Response times	X	n/a	n/a
			Omission errors	X	
			Commission errors	X		

*Significant differences between groups are shown as p < 0.05. No group differences are shown as X*.

* *Response times in incongruent compared to congruent and neutral Flankers*.

### Working Memory

Seventeen studies assessed working memory (Bonnet and Arand, 1995; Randazzo et al., 2000; Rosa and Bonnet, 2000; Vignola et al., 2000; Varkevisser et al., 2007; Haimov et al., 2008; Noh et al., 2012; Joo et al., 2013; Lovato et al., 2013; Siversten et al., 2013; Cellini et al., 2014; Fortier-Brochu and Morin, 2014; Shekleton et al., 2014; Chen et al., 2016; Guo et al., 2017; Khassawneh et al., 2018; Son et al., 2018). Eight of these measured working memory through digit or spatial span backward tests (Randazzo et al., 2000; Vignola et al., 2000; Haimov et al., 2008; Noh et al., 2012; Joo et al., 2013; Lovato et al., 2013; Fortier-Brochu and Morin, 2014; Khassawneh et al., 2018). Four studies used the n-back memory task (Varkevisser et al., 2007; Cellini et al., 2014; Shekleton et al., 2014; Son et al., 2018); two used the memory and search task (Bonnet and Arand, 1995; Rosa and Bonnet, 2000) two used the letter-number sequencing test (Randazzo et al., 2000; Siversten et al., 2013), one used the Corsi block test backward (Noh et al., 2012), one used the nine box maze test to measure spatial and object working memory (Chen et al., 2016) and one the Montreal cognitive assessment battery (Guo et al., 2017).

Nine out of seventeen studies showed significant differences between individuals with insomnia and good sleeper controls on performance (Bonnet and Arand, 1995; Randazzo et al., 2000; Vignola et al., 2000; Haimov et al., 2008; Noh et al., 2012; Joo et al., 2013; Lovato et al., 2013; Cellini et al., 2014; Chen et al., 2016). Of these nine, sleep was objectively impaired in four (Bonnet and Arand, 1995; Haimov et al., 2008; Joo et al., 2013; Cellini et al., 2014). Seven out of seventeen studies did not report information on objective sleep (Randazzo et al., 2000; Varkevisser et al., 2007; Lovato et al., 2013; Siversten et al., 2013; Shekleton et al., 2014; Chen et al., 2016; Guo et al., 2017). With respect to the tasks, performance was consistently impaired in digit and spatial span backward tests, with the exception of two studies (Randazzo et al., 2000; Fortier-Brochu and Morin, 2014). However, Randazzo et al. (2000) found significant impairments in insomnia vs. controls on spatial, but not digit span tasks. The two studies using the two-back memory task failed to find significant differences between individuals with insomnia and controls (Varkevisser et al., 2007; Son et al., 2018). The majority of the studies (five out of eight) which did not report significant differences between subjects with insomnia and controls did not report data on objective sleep (Randazzo et al., 2000; Varkevisser et al., 2007; Siversten et al., 2013; Shekleton et al., 2014; Guo et al., 2017). Findings on working memory are summarised in Table Table 3.

**Table 3:** Comparison of individuals with insomnia and controls on tasks of working memory.

**Author (year)**	***n* Insomnia; controls**	**Task**	**Outcome**	**Group difference**	**Group differences TST**	**Group difference SEI**
Bonnet and Arand, 1995	10; 10	Memory and search task	Correct responses	*p* < 0.05	*p* < 0.05	*p* < 0.05
Cellini et al., 2014	13; 13	N-back memory task	Accuracy	*p* < 0.05	*p* < 0.05	*p* < 0.05
			Number of errors	*p* < 0.05		
			Reaction times	X		
Chen et al., 2016	21;20	Nine box maze test-spatial working memory	Number of errors	*p* < 0.05	n/a	n/a
		Nine box maze test-object working memory	Number of errors	X		
Fortier-Brochu and Morin, 2014	25; 16	Digit span backward	Number of recalled	X	*p* < 0.05	*p* < 0.05
Haimov et al., 2008	35; 64	Digit span backward	Number of recalled	*p* < 0.05	n/a	*p* < 0.05
		Spatial span backward	Number of recalled	*p* < 0.05		
Guo et al., 2017	40;48	Montreal cognitive assessment battery-attention, concentration, and working memory*	Accuracy	X	n/a	n/a
Joo et al., 2013	27; 27	Digit span backward	Number of recalled	*p* < 0.05	*p* < 0.05	*p* < 0.05
Khassawneh et al., 2018	35;54	Spatial working memory test	Number of errors	X	*p* < 0.05	X
			Reaction times	X	
			Strategy	X		
Lovato et al., 2013	49; 49	Double span backward	Number of recalled	*p* < 0.05	n/a	n/a
Noh et al., 2012	20; 20	Digit span backward	Number of recalled	*p* < 0.05	X	X
		Corsi block test backward	Number of recalled	*p* < 0.05		
Randazzo et al., 2000	35; 35	Digit span backward	Number of recalled	X	n/a	n/a
		Spatial span backward	Number of recalled	*p* < 0.05		
		Letter number sequencing test	Correct units in a string	*p* < 0.05		
Rosa and Bonnet, 2000	121; 56	Memory and search task	Correct responses	X	X	X
Shekleton et al., 2014	76; 20	N-back memory task	Reaction times	X	n/a	n/a
Siversten et al., 2013	30; 91	Letter number sequencing test	Correct units in a string	X	n/a	n/a
Son et al., 2018	21;26	Two-back memory task	Reaction times	X	*p* < 0.05	*p* < 0.05
			Accuracy	X	
Varkevisser et al., 2007	39; 20	Two-back memory task	Reaction times	X	n/a	n/a
			Accuracy	X	
Vignola et al., 2000	20; 20	Digit span backward	Number of recalled	*p* < 0.05	X	X

*Significant differences between groups are shown as p < 0.05. No group differences are shown as X*.

### Cognitive Flexibility

Twelve studies reported a measure of cognitive flexibility (Edinger et al., 2000, 2008; Vignola et al., 2000; Altena et al., 2008; Fang et al., 2008; Noh et al., 2012; Joo et al., 2013; Siversten et al., 2013; Fortier-Brochu and Morin, 2014; Shekleton et al., 2014; Guo et al., 2017; Khassawneh et al., 2018). Four studies measured flexibility through switching attention tasks (Edinger et al., 2000, 2008; Shekleton et al., 2014; Khassawneh et al., 2018) and using verbal fluency tasks (Vignola et al., 2000; Siversten et al., 2013; Fortier-Brochu and Morin, 2014). The Wisconsin card sorting test was used in two studies (Vignola et al., 2000; Fang et al., 2008), as well as the controlled oral word association test (Noh et al., 2012; Joo et al., 2013). The trail making test B was used in two studies (Joo et al., 2013; Siversten et al., 2013). Finally, one study used the “visuospatial and executive function” subtest of the Montreal cognitive assessment battery, based on the trail making test B, verbal fluency task and verbal abstraction task (Guo et al., 2017).

Four of twelve studies reported significant differences between individuals with insomnia and good sleepers in some aspects of performance (Edinger et al., 2000, 2008; Noh et al., 2012; Khassawneh et al., 2018). Of these, three also reported between group differences on objective sleep. Specifically, Edinger et al. (2000, 2008) found significant shorter TST and Khassawneh et al. (2018) lower SE in insomnia as compared to controls. Sleep was comparable between the groups in the study of Noh et al. (2012).

Edinger et al. (2000) found that individuals with insomnia showed longer response latency in the switching attention test part III, and the result was replicated by a later study by the same group (Edinger et al., 2008). Similarly, Khassawneh et al. (2018) found slower response latency and higher number of incorrect trials in a similar task. However, Shekleton et al. (2014) replicated the result only in individuals with insomnia and short sleep duration, and no changes to performance was found in subjects with insomnia and normal sleep duration. Fortier-Brochu and Morin (2014) failed to find between group differences in a verbal fluency test. Instead, Noh et al. (2012) found that individuals with insomnia produced less words than controls in the controlled oral association test, reflecting poorer flexibility. However, other researchers failed to replicate these results using similar tests (Altena et al., 2008; Siversten et al., 2013). Performance on the trail making test B (Noh et al., 2012; Joo et al., 2013) and the Wisconsin card sorting test (Vignola et al., 2000; Fang et al., 2008) of individuals with insomnia remained comparable to the controls.

Only three of the eight studies which failed to find significant differences between individuals with insomnia and controls on EFs reported significant differences in TST and SE (Fang et al., 2008; Joo et al., 2013; Fortier-Brochu and Morin, 2014). Vignola et al. (2000) reported no differences on objective TST and SE between those with insomnia and controls, while Altena et al. (2008), Siversten et al. (2013) and Guo et al. (2017) did not provide data on objective sleep measurement. Findings on cognitive flexibility are summarised in Table Table 4.

**Table 4:** Comparison of individuals with insomnia and controls on tasks of cognitive flexibility.

**Author (year)**	***n* Insomnia; controls**	**Task**	**Outcome**	**Group difference**	**Group differences TST**	**Group difference SEI**
Altena et al., 2008	21; 12	Verbal fluency			n/a	n/a
		Letter	Number of words produced	X		
		Category	Number of words produced	X		
Edinger et al., 2000	27; 31	Switching attention task			X	*p* < 0.05
		Part IIIA	Response latency	X		
		Part IIIB	Response latency	*p* < 0.05		
Edinger et al., 2008	79; 84	Switching attention task			X	*p* < 0.05
		Part IIIA	Response latency	*p* < 0.05		
		Part IIIB	Response latency	*p* < 0.05		
Fang et al., 2008	18; 21	Wisconsin card sorting test	Perseverations	X	*p* < 0.05	*p* < 0.05
			Errors	X		
			Conceptual level responses	X		
			Number of categories competed	X		
			Failure to maintaining set	X		
			Learning to learn	X		
Fortier-Brochu and Morin, 2014	25; 16	Verbal fluency	Number of words produced	X	*p* < 0.05	*p* < 0.05
			Set lost errors	X		
			Repetition errors	X		
Guo et al., 2017	40;48	Montreal cognitive assessment- visuospatial and executive function*	Number of correct responses	X	n/a	n/a
Joo et al., 2013	27; 27	Controlled oral word association test	Number of words produced	X	*p* < 0.05	*p* < 0.05
		Trail making test B	Complation time	X		
Khassawneh et al., 2018	35;54	Attention switching task	Response latency	*p* < 0.05	*p* < 0.05	X
			Number of incorrect trials	*p* < 0.05	
			Number of commission errors	X	
Noh et al., 2012	20; 20	Trail making test B	Complation time	X	X	X
		Controlled oral word association test	Number of words produced	*p* < 0.05		
Shekleton et al., 2014	76; 20	Switching attention task			n/a	n/a
		Part IIIA	Response latency	X		
		Part IIIB	Response latency	X		
Siversten et al., 2013	30; 91	Verbal fluency	Number of words produced	X	n/a	n/a
		Trail making test B	Completion time	X		
Vignola et al., 2000	20; 20	Trail making test B	Completion time	X	X	X
		Wisconsin card sorting test	Number of categories competed	X	
			Number of perseverative errors	X		

*Significant differences between groups are shown as p < 0.05. No group differences are shown as X*.

* *Based on Trail making test B, verbal fluency task, and verbal abstraction task*.

### Exploratory Meta-Analysis

#### Inhibitory Control

**Figure 2:**
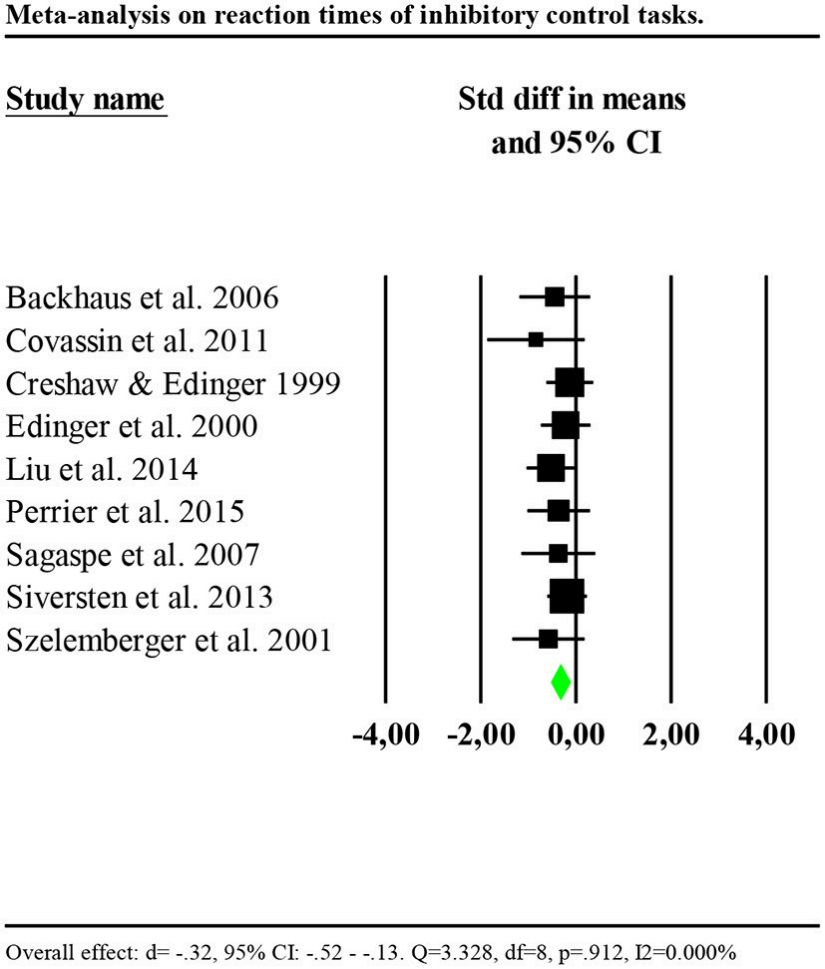
Forest plot of the meta-analysis on reaction times of inhibitory control. Results are presented as standardised mean differences (Std diff) and 95% confidence intervals (CI).

Nine of thirteen studies measuring inhibitory control provided data to compute effect sizes (Crenshaw and Edinger, 1999; Edinger et al., 2000; Szelenberger and Niemcewicz, 2001; Backhaus et al., 2006; Sagaspe et al., 2007; Covassin et al., 2011; Siversten et al., 2013; Liu et al., 2014; Perrier et al., 2015). These reported reaction times as an outcome. Additionally, four of nine studies included a measure of accuracy of the performance (Szelenberger and Niemcewicz, 2001; Backhaus et al., 2006; Sagaspe et al., 2007; Covassin et al., 2011). Thus, we conducted separate analyses for reaction times and accuracy. Reaction times were significantly slower for individuals with insomnia (*n* = 303) than controls (*n* = 355) (*d* = −0.32, 95% CI: −0.52 to −0.13). Heterogeneity statistics were non-significant, showing that the distribution of effects across studies was homogeneous (*Q* = 3.328, df = 8, *p* = 0.912; *I*^2^ = 0.000%). Forest plot of analysis is reported in Figure 2.

We investigated whether the effect size was larger for studies including participants with insomnia and objective sleep impairment (shorter TST or lower SE compared to the controls) by including only these studies in the analysis (Edinger et al., 2000; Backhaus et al., 2006; Covassin et al., 2011; Liu et al., 2014; Perrier et al., 2015). Results showed a significant and larger effect (*d* = −0.41, 95% CI: −0.69 to −0.13). Heterogeneity statistics were non-significant (*Q* = 1.436, df = 4, *p* = 0.838; *I*^2^ = 0.000%). In contrast, including only studies which failed to find significant differences between insomnia and control groups or missed to report information on objective sleep (Crenshaw and Edinger, 1999; Szelenberger and Niemcewicz, 2001; Sagaspe et al., 2007; Siversten et al., 2013), results showed a smaller effect (*d* = −0.24, 95% CI: −0.51 to −0.03), with low and non-significant heterogeneity (*Q* = 1.144, df = 3, *p* = 0.766; *I*^2^ = 0.000%).

**Figure 3:**
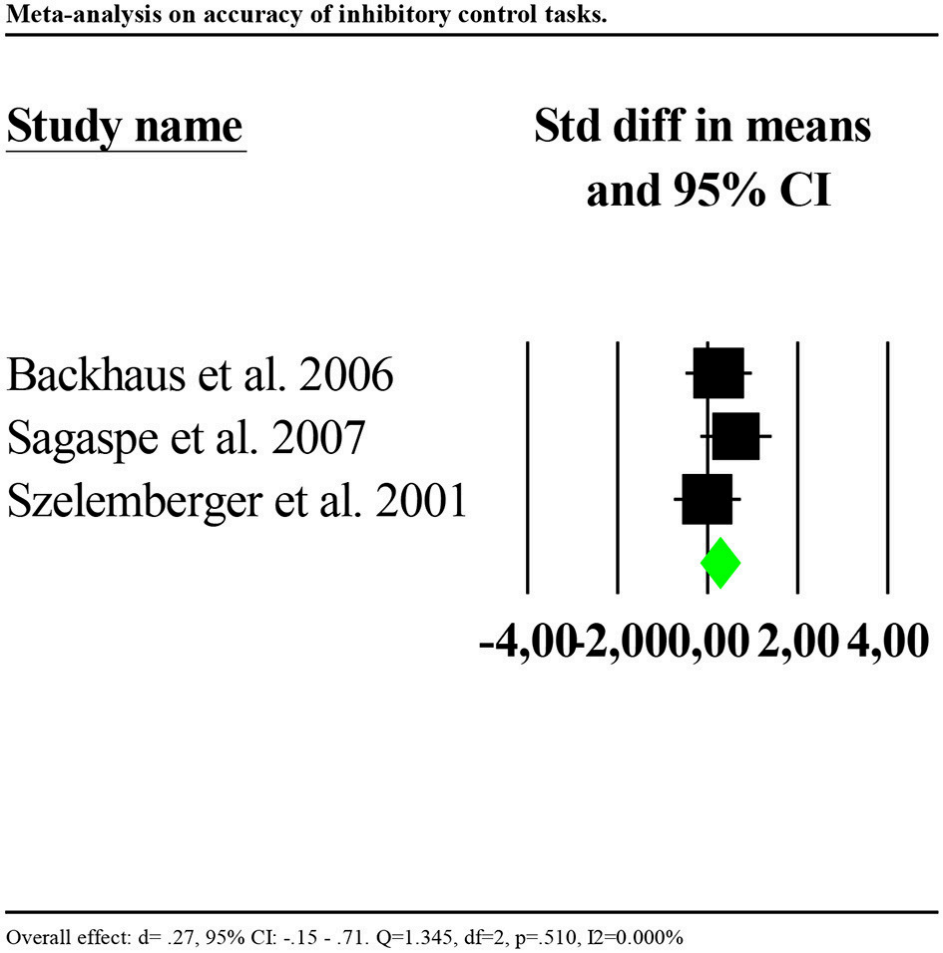
Forest plot of the meta-analysis on accuracy of inhibitory control (outlier removed). Results are presented as standardised mean differences (Std diff) and 95% confidence intervals (CI).

Results on accuracy showed that the performance of individuals with insomnia was significantly more accurate than that of controls (*d* = 0.504, 95% CI: 0.082 to 0.925). Nevertheless, effects distribution was significantly heterogenous between studies (*Q* = 19.162, df = 3, *p*<*0.0*01; *I*^2^ = 84.344%). We repeated the analysis removing one potential outlier (Covassin et al., 2011, *d* = 0.4.21, 95% CI: 2.38–5.85). Results showed smaller and no longer significant effect (*d* = 0.27, 95% CI: −0.15 to 0.71). Heterogeneity tests were low and no longer significant, reflecting a homogeneous distribution of the effects across studies (*Q* = 1.345, df = 2, *p* = 0.510; *I*^2^ = 0.000%). Forest plot of the analysis is reported in Figure 3. Given the small number of studies, we were limited in performing further sensitivity analyses.

### Working Memory

**Figure 4:**
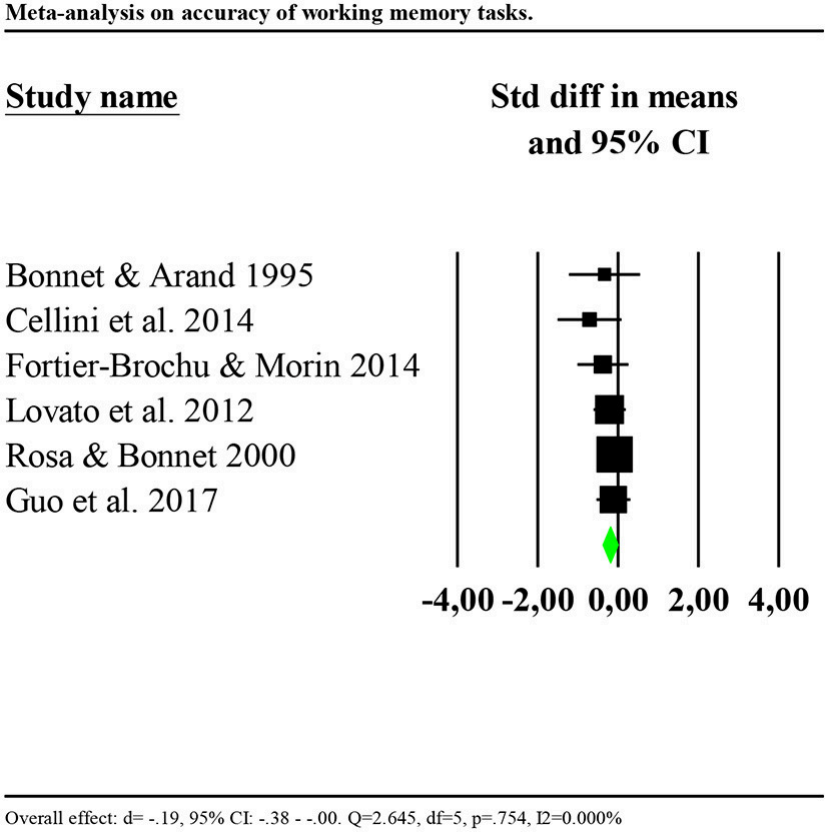
Forest plot of the meta-analysis on accuracy of working memory (outlier removed). Results are presented as standardised mean differences (Std diff) and 95% confidence intervals (CI).

Eight studies reported data of accuracy of the performance (Bonnet and Arand, 1995; Rosa and Bonnet, 2000; Lovato et al., 2013; Cellini et al., 2014; Fortier-Brochu and Morin, 2014; Guo et al., 2017; Khassawneh et al., 2018; Son et al., 2018). Only one study reported data for reaction times (Cellini et al., 2014). Consequently, we performed the analysis for accuracy only. Analysis showed no significant results (*d* = −0.034, 95% CI: −0.211 to 0.144) and high heterogeneity (*Q* = 42.678, df = 6, *p* < 0.001; *I*^2^ = 85.941%). We repeated the analysis removing one potential outlier (Khassawneh et al., 2018, *d* = −1.161, 95% CI: −1.93 to −0.98). Results showed a small significant effect (*d* = −0.19, 95% CI: −0.38 to −0.00). Heterogeneity statistics were not significant (*Q* = 2.645, df = 5, *p* = 0.754; *I*^2^ = 0.000%). Forest plot of this analysis is reported in Figure 4.

We investigated whether the effect was larger for studies including participants with insomnia and objective sleep impairment (shorter TST or lower SE compared to the controls) by including only these in the analysis (Bonnet and Arand, 1995; Cellini et al., 2014; Fortier-Brochu and Morin, 2014). Results showed a significant and larger effect (*d* = −0.46, 95% CI: −0.03 to −0.89). Heterogeneity statistics were non-significant (*Q* = 0.525, df = 2, *p* = 0.769; *I*^2^ = 0.000%). In contrast, including only studies which failed to find significant differences between insomnia and control groups, or missed to report information on objective sleep, showed a smaller and non-significant effect (*d* = −0.13, 95% CI: −0.08 to 0.34), with low and non-significant heterogeneity (*Q* = 0.240, df = 2, *p* = 0.887; *I*^2^ = 0.000%).

### Cognitive Flexibility

**Figure 5:**
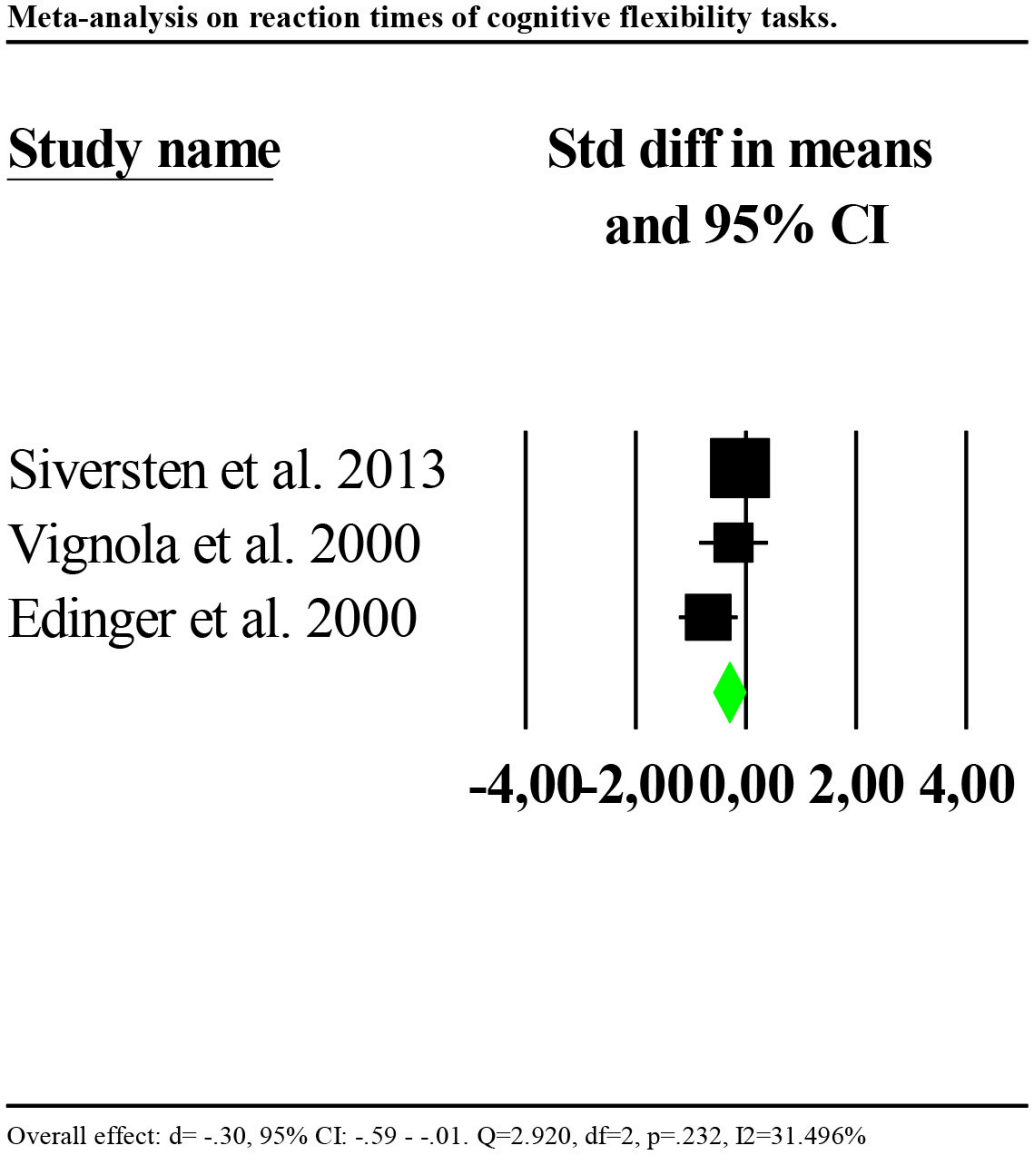
Forest plot of the meta-analysis on reaction times of cognitive flexibility (outlier removed). Results are presented as standardised mean differences (Std diff) and 95% confidence intervals (CI).

Four studies reported reaction times (Edinger et al., 2000; Vignola et al., 2000; Siversten et al., 2013; Khassawneh et al., 2018) and four accuracy (Fang et al., 2008; Fortier-Brochu and Morin, 2014; Guo et al., 2017; Khassawneh et al., 2018) as outcomes. Consequently, we ran separate analyses for reaction times and accuracy. Reaction times were significantly slower for individuals with insomnia as compared to controls (*d* = −0.77, 95% CI: −1.03 to −0.51). Nevertheless, the distribution of effects was highly and significantly heterogenous between studies (*Q* = 54.954, df = 3, *p* < 0.001; *I*^2^ = 94.541). We repeated the analysis removing one potential outlier (Khassawneh et al., 2018, *d* = −2.689, 95% CI: −3.26 to −2.10). Results showed a significant effect (*d* = −0.30, 95% CI: −0.59 to −0.01). Heterogeneity tests were low and no longer significant (*Q* = 2.920, df = 2, *p* = 0.232; *I*^2^ = 31.496). Forest plot of this analysis is reported in Figure 5.

**Figure 6:**
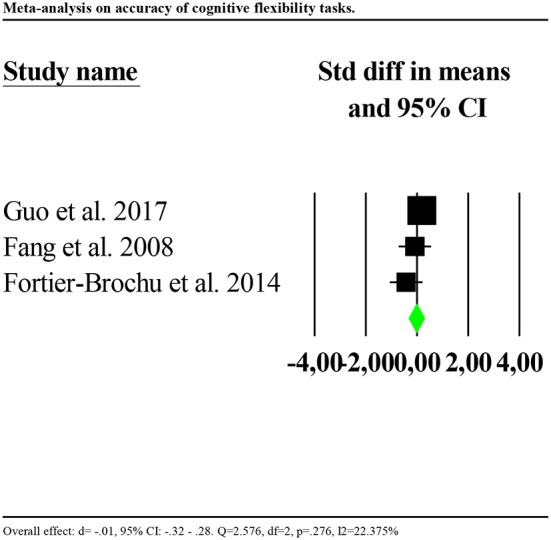
Forest plot of the meta-analysis on accuracy of cognitive flexibility (outlier removed). Results are presented as standardised mean differences (Std diff) and 95% confidence intervals (CI).

Accuracy was significantly poorer for individuals with insomnia than controls (*d* = −0.602, 95% CI: −0.873 to −0.330). Again, the distribution of effects was highly and significantly heterogenous between studies (*Q* = 67.580, df = 3, *p* < 0.001; *I*^2^ = 95.561). We repeated the analysis removing one potential outlier (Khassawneh et al., 2018, *d* = −2.733, 95% CI: −3.31 to −2.14). Results showed no significant effects (*d* = −0.017, 95% CI: −0.32 to 0.28). Heterogeneity tests were low and no longer significant (*Q* = 2.576, df = 2, *p* = 0.276; *I*^2^ = 22.375). Forest plot of this analysis is reported in Figure 6.

In addition, since three studies reported the same outcome using the same task (the number of words produced in the verbal fluency task) (Altena et al., 2008; Siversten et al., 2013; Fortier-Brochu and Morin, 2014), we ran a separate analysis on verbal fluency tasks. Results showed a slight and marginally significant tendency toward a better performance for individuals with insomnia as compared to controls (*d* = 0.313, 95% CI: −0.000 to 0.617). Heterogeneity tests were low and non-significant (*Q* = 3.371, df = 2, *p* = 0.176; *I*^2^ = 42.380).

### Risk of Bias

**Figure 7:**
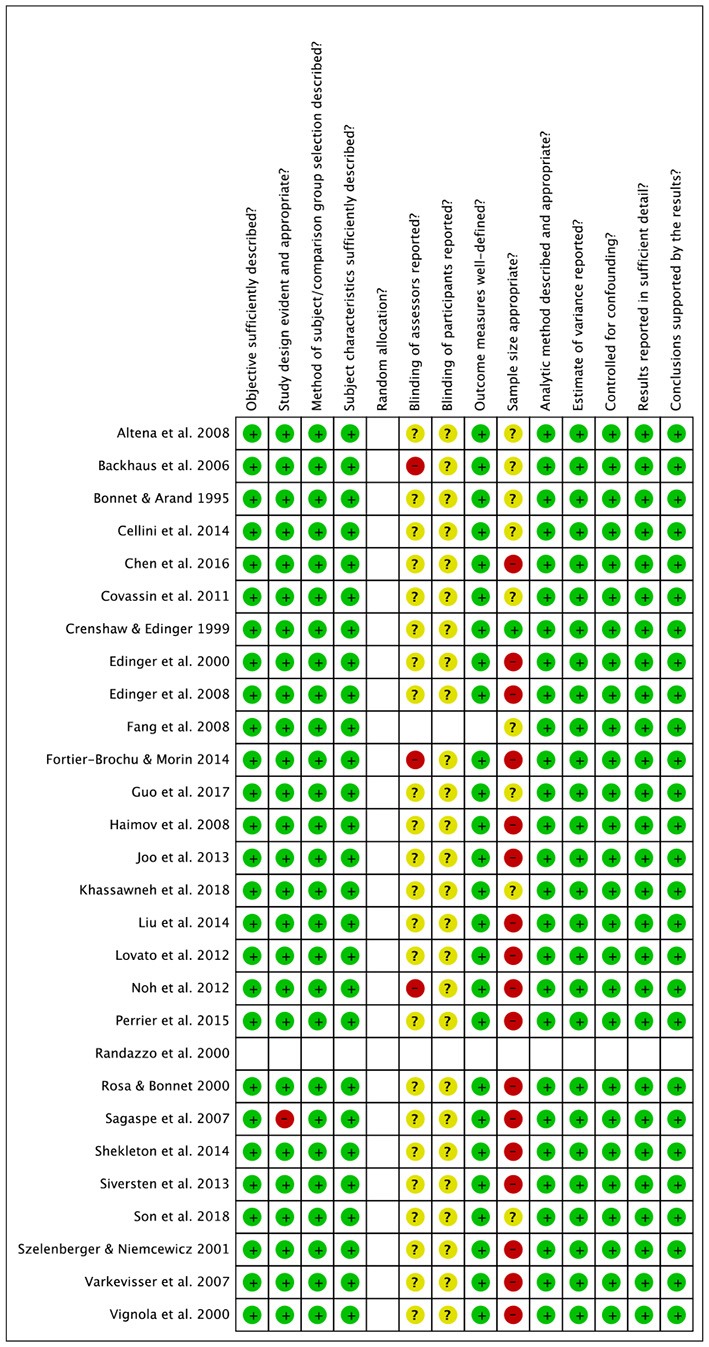
Risk of bias summary.

**Figure 8:**
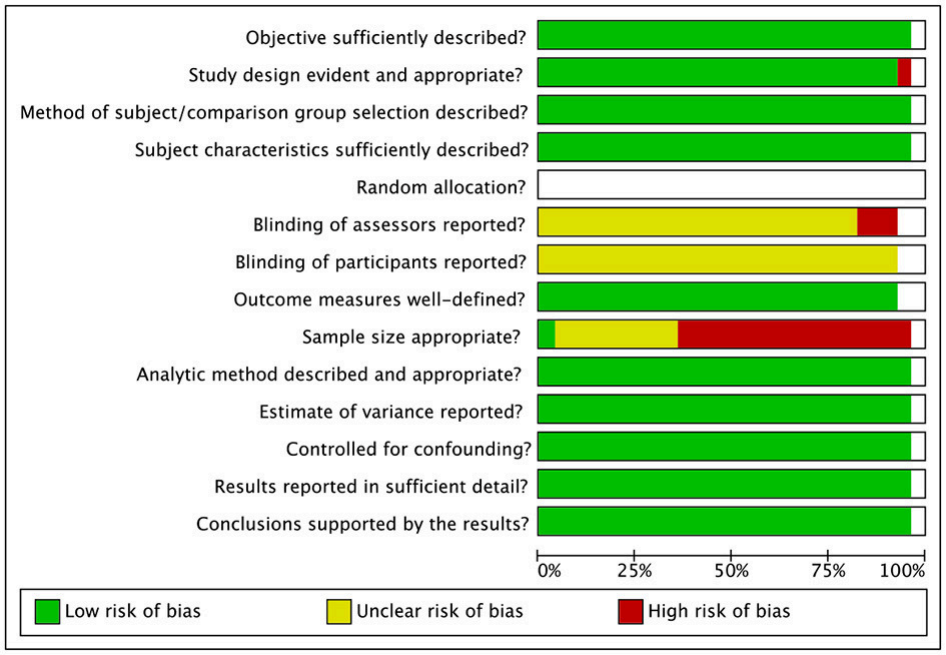
Risk of bias graph.

Risk of bias assessment ratings are reported in Figures 7, 8. In general, studies were judged as having low risk of bias. Small sample size of individual studies emerged as a potential source of bias. Blinding of outcome assessors and participants were judged as two areas of partially biased.

## Discussion

The present systematic review aimed to examine the presence and magnitude of inhibitory control, working memory and cognitive flexibility impairments in individuals with insomnia vs. controls. Using combined narrative synthesis and meta-analysis, we gathered evidence supporting impaired functioning in several aspects of EFs in insomnia. Due to few studies reporting data to compute effect sizes, small sample sizes and high heterogeneity of effects distribution, results from the present review should be interpreted carefully.

Meta-analytic findings support the presence of impaired performance of individuals with insomnia as compared to controls in reaction time-based tasks assessing inhibitory control and cognitive flexibility, with effects sizes ranging from small to moderate in magnitude. In contrast, accuracy rates (i.e., correct responses), were found intact in insomnia with respect to inhibitory control and cognitive flexibility tasks but impaired in working memory tasks. The present work advances knowledge on cognitive functioning in insomnia, updating previous meta-analytic work (Fortier-Brochu et al., 2012) and providing a more detailed assessment of executive processes. In particular, our findings corroborate the results of Fortier-Brochu's review with respect to insomnia-related working memory deficits and provide further evidence for inhibitory control and cognitive flexibility impairments. This advancement is due to the great number of studies (*n* = 14) published on this topic after Fortier-Brochu's meta-analysis.

### Objective Sleep Impairments

To examine the hypothesis that EFs are impaired only in insomnia with objective sleep impairments (i.e., shortened TST and/or reduced SE; Fernandez-Mendoza et al., 2010; Vgontzas et al., 2013), which may explain conflicting study findings (Shekleton et al., 2010), we extracted data on the difference between individuals with insomnia and good sleepers on TST and SE. Consequently, we investigated the relationship between presence of EFs impairments and objective sleep impairments. Both narrative synthesis and meta-analysis highlighted that the magnitude of EFs impairments was larger in studies including participants with insomnia and objective shorter TST and/or lower SE as compared to the controls rather than in studies which failed to report significant between group differences in objective sleep or did not report this information. More specifically, this hypothesis was statistically verified for reaction times in inhibitory control tasks and accuracy rates in working memory tasks. Due to the small number of studies, it was not possible to statistically test the objective sleep hypothesis for cognitive flexibility tasks. Nevertheless, these findings could have important implications for treatment development; in particular, tailoring treatment for varying needs, and differential effects of treatment on populations of diverse clinical characteristics.

These results, therefore, are apparently consistent the hypothesis that higher order neuropsychological functions may be quite preserved in individuals with insomnia with normal sleep and provide partial evidence for the theory that only the phenotype of insomnia with objective sleep impairment is associated with worst objective neuropsychological deficits (Fernandez-Mendoza et al., 2010; Vgontzas et al., 2013). This is also in line with sleep deprivation literature demonstrating an impairment of EFs after sleep loss (Nilsson et al., 2005; Couyoumdjian et al., 2010; Martella et al., 2011).

Nevertheless, due to the small number of studies included in the meta-analytic calculations, these results should be interpreted carefully. Larger trials conducted in both insomnia with normal sleep and objective sleep impairments and including both reaction times and accuracy indices are needed to confirm our findings. Moreover, as discussed in detail below, it is desirable for future studies to elucidate the potentially differential effects of sleep quality and sleep quantity on EFs, that we were unable to investigate due to the small number of studies available.

### Cognitive Tests

Many neuropsychological tests used to assess EF, such as the trail making test and the Wisconsin card sorting test, have been validated to assess deficits of large magnitude in patients with brain injuries or neurological disorders and are therefore unlikely to detect minor impairment experienced by those without brain injury, such as those with insomnia. Although deficits in the trail making test are reported in samples with severe psychiatric disorder like major depression or bipolar disorder (Pattanayak et al., 2012; Cotrena et al., 2016), other neuropsychological tests such as the colour-word interference tests, backward memory span tasks, and switching attention tasks may be more sensitive to detecting less severe deficits such as those affecting subjects with insomnia. In fact, individuals with insomnia consistently showed impaired performance in these tasks. Thus, future studies of EFs in insomnia samples would benefit from considering for test sensitivity. Importantly, future research on EFs in insomnia would benefit from including tasks with reasonably similar paradigms (i.e., similar instructions and procedure of assessment, similar outcomes). The presence of varied tasks used to assess the same cognitive functions (e.g., 5 different tasks on 13 studies to assess inhibitory control), is a potential source of bias. It would be therefore important for insomnia research to standardise cognitive assessment procedures in order to reduce variability in studies' methodology.

### Age

A variable that may have potentially influenced the results is age. It has been suggested that different executive processes decline with increasing age, and this decline has been associated with changes in brain areas including frontal lobes and their connections with other brain areas (e.g., Jurado and Rosselli, 2007 for a review). In our sample, about the 60% of the studies reported significant differences between insomnia those with insomnia and controls on EFs, without remarkable differences between studies conducted in adult and elderly populations. Only two studies were conducted on young adults (Covassin et al., 2011; Cellini et al., 2014), and both reported significantly impaired performance in those with insomnia compared to controls respectively on tasks of inhibitory control and working memory. However, the studies were conducted by the same research group and this may potentially limit the generalization of the results. Moreover, differences in age between those with insomnia and controls were limited, with the exception of one study including participants with insomnia 10 years older than controls (Khassawneh et al., 2018). In summary, age is a variable that should be further investigated in cognitive studies conducted in insomnia. Also, there is a dearth of literature on EFs in elderly and young adults with insomnia.

### Limitations

Although the current review provides a comprehensive synthesis of the literature concerning EFs and insomnia, there are several limitations which should be acknowledged. Consistent with Cochrane guidelines for systematic reviews (Higgins and Green, 2011), we searched three databases. It is possible that searching additional databases (e.g., EMBASE) may have produced additional studies, although our other approaches (e.g., searching reference lists of included papers) make this less likely. Also, in this review, we adopted the definition of EFs based on inhibitory control, working memory, and cognitive flexibility (Miyake et al., 2000; Diamond, 2013), a very influential classification recently used in the context of insomnia research (Fernandez-Mendoza et al., 2010; Vgontzas et al., 2013). However, as aforementioned, controversies and divergences on the definition and conceptualisation of EFs, with many other models of EFs previously proposed (see Gratton et al., 2017 for a review); thus, future systematic reviews integrating those models may highlight further important findings and achieve different conclusions. Additionally, the identification and categorisation of executive tasks used in this review may also present some limitations. We decided to base the identification and categorisation of the tests on recent literature (Fortier-Brochu et al., 2012; Diamond, 2013; Snyder et al., 2015) whilst other researchers in the field have used different classifications (Shekleton et al., 2010). Thus, it is possible that slightly different results may emerge due to the use of different classifications of the tasks. Also, we decided to extract data on objective sleep derived from both actigraphy and polysomnography and to consider these measures in the same analysis. Although both measures allow to objectively assess TST and SE, the two measures are based on different psychophysiological processes. Actigraphy is based on body movements while polysomnography is based on a combination of electroencephalogram, electrooculogram and electromyogram, which also permits derivation of sleep architecture information. It has been observed in validation studies, that TST and SE derived from the two methods of assessment correlate (*r* = 0.87 for TST and *r* = 0.56 for SE; e.g., Lichstein et al., 2006; Williams et al., 2018). However, contrasting evidence also suggested limited validity of actigraphy when compared to polysomnography (e.g., Sànchez-Ortun o et al., 2010; Natale et al., 2014). We decided to pool them in the same analysis to reach a sufficient number of effect sizes to analyse. However, given contrasting literature, the two measures may be ideally considered in different analyses in future studies with larger samples. Relatedly, we focussed on TST and SE as two measures reflecting night-time symptoms of insomnia (i.e., longer time needed to fall asleep, frequent and long nocturnal awakenings). While TST is a measure of sleep duration, SE is generally considered a measure of general sleep quality. Thus, the two measures may potentially have differential effects on EFs. Given the limited number of studies included in this review, we were limited in investigating the differential effect of SE and TST on EFs in meta-analysis. Again, it is important for future studies to include objective measures of SE and TST to better elucidate their effects on EFs performance in insomnia.

### Conclusions

The study of EFs has dramatically increased in recent years in the context of mental health. However, EFs are still under-investigated in insomnia. To make a comparison, a recent and already cited review of meta-analytic literature (Snyder et al., 2015) found that ten meta-analyses were conducted studying EFs in bipolar, eight in schizophrenia, seven in substance use, four in anxiety and two in depressive disorders, while only one meta-analysis was conducted in samples with insomnia (Fortier-Brochu et al., 2012). This discrepancy is particularly surprising given the well documented detrimental effects of insomnia on daytime variables in which EFs may play an important role, such as memory, attention and concentration and emotion regulation (Kyle et al., 2013; Harris et al., 2015; Cellini, 2016) and the consideration of insomnia as a transdiagnostic process across mental disorders (Harvey, 2009; Dolsen et al., 2014). Future studies with comparable procedures of neuropsychological assessment are needed to clarify the nature and strength of the association between insomnia and EFs deficits. Such standardisation of assessments could lead to important clinical and research applications. Interventional studies, aiming at investigating whether EFs impairments in insomnia are reversible are needed. Randomised controlled trials of cognitive behavioural therapy for insomnia showed promising results on core EFs (Herbert et al., 2018). Nevertheless, replication studies are needed to consolidate these results and evaluate the cost-effectiveness of such treatment approaches on factors including healthcare utilisation and burden of illness (e.g., absenteeism, workplace errors, quality of life). Additionally, it is yet to be explored whether improvement in EFs after insomnia treatment is associated with ameliorate subjective functioning (Kyle et al., 2013; Ballesio et al., 2018). Future research is particularly needed on elderly and young adults which are under-investigated populations. Moreover, researchers should consider variables that are largely neglected in this field, including, besides objective sleep, the time of testing, that may influence study results due the fluctuations of circadian rhythm (e.g., Varkevisser and Kerkhof, 2005).

## Author Contributions

AB run the literature searches, extracted qualitative and quantitative data, assessed the risk of bias, run the analyses, and wrote the draft of the manuscript. MRJVA screened the abstracts and full-texts, assessed the risk of bias, and revised the draft. SK, FF, and CL provided intellectual and analytical advices, supervised the work, and revised the manuscript.

### Conflict of Interest Statement

The authors declare that the research was conducted in the absence of any commercial or financial relationships that could be construed as a potential conflict of interest.
