# Supplementary material for: Executive Functions in Insomnia Disorder: A Systematic Review and Exploratory Meta-Analysis
Source: Front Psychol. 2019 Jan 30;10:101. doi: 10.3389/fpsyg.2019.00101 (PMC6363670; doi:10.3389/fpsyg.2019.00101)
Supplement: Supplementary file 1 [file Table_1.DOCX]

***Executive function in insomnia disorder: a systematic review and exploratory meta-analysis. Document S1*.**

**Table of content**

1. Excluded studies and reason for exclusion;
2. PRISMA Checklist;
3. **Excluded studies and reason for exclusion**

*Not including a measure of inhibitory control, working memory and cognitive flexibility*

1. Bastien CH, St-Jean G, Morin CM, Turcotte I, Carrier J. Chronic psychophysiological insomnia: hyperarousal and/or inhibition deficits? An ERPs investigation. Sleep 2008;31:887-98.
2. [Contreras-González N](http://www.ncbi.nlm.nih.gov/pubmed/?term=Contreras-Gonz%C3%A1lez%20N%5BAuthor%5D&cauthor=true&cauthor_uid=26923575), [Téllez-Alanís B](http://www.ncbi.nlm.nih.gov/pubmed/?term=T%C3%A9llez-Alan%C3%ADs%20B%5BAuthor%5D&cauthor=true&cauthor_uid=26923575), [Haro R](http://www.ncbi.nlm.nih.gov/pubmed/?term=Haro%20R%5BAuthor%5D&cauthor=true&cauthor_uid=26923575), [Jiménez-Correa U](http://www.ncbi.nlm.nih.gov/pubmed/?term=Jim%C3%A9nez-Correa%20U%5BAuthor%5D&cauthor=true&cauthor_uid=26923575), [Poblano A](http://www.ncbi.nlm.nih.gov/pubmed/?term=Poblano%20A%5BAuthor%5D&cauthor=true&cauthor_uid=26923575). Executive dysfunction in patients with chronic primary insomnia treated with clonazepam. Neurol Res 2015;37:1047-53.
3. [Cortoos A](http://www.ncbi.nlm.nih.gov/pubmed/?term=Cortoos%20A%5BAuthor%5D&cauthor=true&cauthor_uid=23541997), [De Valck E](http://www.ncbi.nlm.nih.gov/pubmed/?term=De%20Valck%20E%5BAuthor%5D&cauthor=true&cauthor_uid=23541997), [Pattyn N](http://www.ncbi.nlm.nih.gov/pubmed/?term=Pattyn%20N%5BAuthor%5D&cauthor=true&cauthor_uid=23541997), [Mairesse O](http://www.ncbi.nlm.nih.gov/pubmed/?term=Mairesse%20O%5BAuthor%5D&cauthor=true&cauthor_uid=23541997), [Cluydts R](http://www.ncbi.nlm.nih.gov/pubmed/?term=Cluydts%20R%5BAuthor%5D&cauthor=true&cauthor_uid=23541997). Excitatory vs inhibitory impairments in insomnia patients:an ERP study. J Int Psychophysiol 2014;62-9.
4. [Huang Z](http://www.ncbi.nlm.nih.gov/pubmed/?term=Huang%20Z%5BAuthor%5D&cauthor=true&cauthor_uid=22424903), [Zhan S](http://www.ncbi.nlm.nih.gov/pubmed/?term=Zhan%20S%5BAuthor%5D&cauthor=true&cauthor_uid=22424903), [Li N](http://www.ncbi.nlm.nih.gov/pubmed/?term=Li%20N%5BAuthor%5D&cauthor=true&cauthor_uid=22424903), [Ding Y](http://www.ncbi.nlm.nih.gov/pubmed/?term=Ding%20Y%5BAuthor%5D&cauthor=true&cauthor_uid=22424903), [Wang Y](http://www.ncbi.nlm.nih.gov/pubmed/?term=Wang%20Y%5BAuthor%5D&cauthor=true&cauthor_uid=22424903). Abnormal recovery function of somatosensory evoked potentials in patients with primary insomnia. Psychiatry Res 2012;198:463-7.
5. [Kales A](http://www.ncbi.nlm.nih.gov/pubmed/?term=Kales%20A%5BAuthor%5D&cauthor=true&cauthor_uid=6622623), [Caldwell AB](http://www.ncbi.nlm.nih.gov/pubmed/?term=Caldwell%20AB%5BAuthor%5D&cauthor=true&cauthor_uid=6622623), [Soldatos CR](http://www.ncbi.nlm.nih.gov/pubmed/?term=Soldatos%20CR%5BAuthor%5D&cauthor=true&cauthor_uid=6622623), [Bixler EO](http://www.ncbi.nlm.nih.gov/pubmed/?term=Bixler%20EO%5BAuthor%5D&cauthor=true&cauthor_uid=6622623), [Kales JD](http://www.ncbi.nlm.nih.gov/pubmed/?term=Kales%20JD%5BAuthor%5D&cauthor=true&cauthor_uid=6622623). Biopsychobehavioral correlates of insomnia. II. Pattern specificity and consistency with the Minnesota Multiphasic Personality Inventory. Psychosom Med 1983;45:341-56.
6. [McCracken LM](http://www.ncbi.nlm.nih.gov/pubmed/?term=McCracken%20LM%5BAuthor%5D&cauthor=true&cauthor_uid=21539701), [Williams JL](http://www.ncbi.nlm.nih.gov/pubmed/?term=Williams%20JL%5BAuthor%5D&cauthor=true&cauthor_uid=21539701), [Tang NK](http://www.ncbi.nlm.nih.gov/pubmed/?term=Tang%20NK%5BAuthor%5D&cauthor=true&cauthor_uid=21539701). Psychological flexibility may reduce insomnia in persons with chronic pain: a preliminary retrospective study. Pain Med 2011;12:904-12.
7. [Stoffers D](http://www.ncbi.nlm.nih.gov/pubmed/?term=Stoffers%20D%5BAuthor%5D&cauthor=true&cauthor_uid=24285642), [Altena E](http://www.ncbi.nlm.nih.gov/pubmed/?term=Altena%20E%5BAuthor%5D&cauthor=true&cauthor_uid=24285642), [van der Werf YD](http://www.ncbi.nlm.nih.gov/pubmed/?term=van%20der%20Werf%20YD%5BAuthor%5D&cauthor=true&cauthor_uid=24285642), [Sanz-Arigita EJ](http://www.ncbi.nlm.nih.gov/pubmed/?term=Sanz-Arigita%20EJ%5BAuthor%5D&cauthor=true&cauthor_uid=24285642), [Voorn TA](http://www.ncbi.nlm.nih.gov/pubmed/?term=Voorn%20TA%5BAuthor%5D&cauthor=true&cauthor_uid=24285642), [Astill RG](http://www.ncbi.nlm.nih.gov/pubmed/?term=Astill%20RG%5BAuthor%5D&cauthor=true&cauthor_uid=24285642) et al. The caudate: a key node in the network imbalance of insomnia? Brain 2014;610-20.

*Not including a group with standardised diagnosis of insomnia*

1. [Boland EM](http://www.ncbi.nlm.nih.gov/pubmed/?term=Boland%20EM%5BAuthor%5D&cauthor=true&cauthor_uid=26474660), [Stange JP](http://www.ncbi.nlm.nih.gov/pubmed/?term=Stange%20JP%5BAuthor%5D&cauthor=true&cauthor_uid=26474660), [Molz Adams A](http://www.ncbi.nlm.nih.gov/pubmed/?term=Molz%20Adams%20A%5BAuthor%5D&cauthor=true&cauthor_uid=26474660), [LaBelle DR](http://www.ncbi.nlm.nih.gov/pubmed/?term=LaBelle%20DR%5BAuthor%5D&cauthor=true&cauthor_uid=26474660), [Ong ML](http://www.ncbi.nlm.nih.gov/pubmed/?term=Ong%20ML%5BAuthor%5D&cauthor=true&cauthor_uid=26474660), [Hamilton JL](http://www.ncbi.nlm.nih.gov/pubmed/?term=Hamilton%20JL%5BAuthor%5D&cauthor=true&cauthor_uid=26474660) et al. Associations between sleep disturbance, cognitive functioning and work disability in bipolar disorder. Psychiatry Res 2015;230:567-74.
2. [Edwards RR](http://www.ncbi.nlm.nih.gov/pubmed/?term=Edwards%20RR%5BAuthor%5D&cauthor=true&cauthor_uid=19168380), [Grace E](http://www.ncbi.nlm.nih.gov/pubmed/?term=Grace%20E%5BAuthor%5D&cauthor=true&cauthor_uid=19168380), [Peterson S](http://www.ncbi.nlm.nih.gov/pubmed/?term=Peterson%20S%5BAuthor%5D&cauthor=true&cauthor_uid=19168380), [Klick B](http://www.ncbi.nlm.nih.gov/pubmed/?term=Klick%20B%5BAuthor%5D&cauthor=true&cauthor_uid=19168380), [Haythornthwaite JA](http://www.ncbi.nlm.nih.gov/pubmed/?term=Haythornthwaite%20JA%5BAuthor%5D&cauthor=true&cauthor_uid=19168380), [Smith MT](http://www.ncbi.nlm.nih.gov/pubmed/?term=Smith%20MT%5BAuthor%5D&cauthor=true&cauthor_uid=19168380). Sleep continuity and architecture: association with pain-inhibitory processes in patients with temporomandibular joint disorder. Eur J Pain 2009;1043-7.
3. [Fernandez-Mendoza J](http://www.ncbi.nlm.nih.gov/pubmed/?term=Fernandez-Mendoza%20J%5BAuthor%5D&cauthor=true&cauthor_uid=20394314), [Calhoun S](http://www.ncbi.nlm.nih.gov/pubmed/?term=Calhoun%20S%5BAuthor%5D&cauthor=true&cauthor_uid=20394314), [Bixler EO](http://www.ncbi.nlm.nih.gov/pubmed/?term=Bixler%20EO%5BAuthor%5D&cauthor=true&cauthor_uid=20394314), [Pejovic S](http://www.ncbi.nlm.nih.gov/pubmed/?term=Pejovic%20S%5BAuthor%5D&cauthor=true&cauthor_uid=20394314), [Karataraki M](http://www.ncbi.nlm.nih.gov/pubmed/?term=Karataraki%20M%5BAuthor%5D&cauthor=true&cauthor_uid=20394314), [Liao D](http://www.ncbi.nlm.nih.gov/pubmed/?term=Liao%20D%5BAuthor%5D&cauthor=true&cauthor_uid=20394314), [Vela-Bueno A](http://www.ncbi.nlm.nih.gov/pubmed/?term=Vela-Bueno%20A%5BAuthor%5D&cauthor=true&cauthor_uid=20394314) et al. Insomnia with objective short sleep duration is associated with deficits in neuropsychological performance: a general population study. Sleep 2010;33:459-65.
4. Gamaldo CE, Gamaldo A, Creighton J, Salas RE, Selnes OA, David PM et al. Sleep and cognition in HIV+color: a multi-method approach. J Acquir Immune Defic Syndr 2013;63: doi:10.1097/QAI.0b013e31829d63ab.
5. [Hovland A](http://www.ncbi.nlm.nih.gov/pubmed/?term=Hovland%20A%5BAuthor%5D&cauthor=true&cauthor_uid=23347472), [Pallesen S](http://www.ncbi.nlm.nih.gov/pubmed/?term=Pallesen%20S%5BAuthor%5D&cauthor=true&cauthor_uid=23347472), [Hammar A](http://www.ncbi.nlm.nih.gov/pubmed/?term=Hammar%20A%5BAuthor%5D&cauthor=true&cauthor_uid=23347472), [Hansen AL](http://www.ncbi.nlm.nih.gov/pubmed/?term=Hansen%20AL%5BAuthor%5D&cauthor=true&cauthor_uid=23347472), [Thayer JF](http://www.ncbi.nlm.nih.gov/pubmed/?term=Thayer%20JF%5BAuthor%5D&cauthor=true&cauthor_uid=23347472), [Sivertsen B](http://www.ncbi.nlm.nih.gov/pubmed/?term=Sivertsen%20B%5BAuthor%5D&cauthor=true&cauthor_uid=23347472) et al. Subjective sleep quality in relation to inhibition and heart rate variabilità in patients with panic disorder. J Affect Disord 2013;150:152-5.
6. Lundh LG, Froding A, Gyllenhammar L, Broman JE, Hetta J. Cognitive bias and memory performance in patients with persistent insomnia. Scandinavian Journal of Behaviour Therapy 1997;26:27-35.
7. Mendelson WB, Garnett D, Gilin JC, Weingartner H. The experience of insomnia and daytime and nighttime functioning. Psychiatry Res 1984;12:235-250.
8. [Naismith SL](http://www.ncbi.nlm.nih.gov/pubmed/?term=Naismith%20SL%5BAuthor%5D&cauthor=true&cauthor_uid=20354239), [Rogers NL](http://www.ncbi.nlm.nih.gov/pubmed/?term=Rogers%20NL%5BAuthor%5D&cauthor=true&cauthor_uid=20354239), [Hickie IB](http://www.ncbi.nlm.nih.gov/pubmed/?term=Hickie%20IB%5BAuthor%5D&cauthor=true&cauthor_uid=20354239), [Mackenzie J](http://www.ncbi.nlm.nih.gov/pubmed/?term=Mackenzie%20J%5BAuthor%5D&cauthor=true&cauthor_uid=20354239), [Norrie LM](http://www.ncbi.nlm.nih.gov/pubmed/?term=Norrie%20LM%5BAuthor%5D&cauthor=true&cauthor_uid=20354239), [Lewis SJ](http://www.ncbi.nlm.nih.gov/pubmed/?term=Lewis%20SJ%5BAuthor%5D&cauthor=true&cauthor_uid=20354239). Sleep well, think well: sleep-wake disturbance in mild cognitive impairment. J Geriatr Psychiatry Neurol 2010;23:123-30.
9. [Naismith SL](http://www.ncbi.nlm.nih.gov/pubmed/?term=Naismith%20SL%5BAuthor%5D&cauthor=true&cauthor_uid=21435728), [Rogers NL](http://www.ncbi.nlm.nih.gov/pubmed/?term=Rogers%20NL%5BAuthor%5D&cauthor=true&cauthor_uid=21435728), [Lewis SJ](http://www.ncbi.nlm.nih.gov/pubmed/?term=Lewis%20SJ%5BAuthor%5D&cauthor=true&cauthor_uid=21435728), [Terpening Z](http://www.ncbi.nlm.nih.gov/pubmed/?term=Terpening%20Z%5BAuthor%5D&cauthor=true&cauthor_uid=21435728), [Ip T](http://www.ncbi.nlm.nih.gov/pubmed/?term=Ip%20T%5BAuthor%5D&cauthor=true&cauthor_uid=21435728), [Diamond K](http://www.ncbi.nlm.nih.gov/pubmed/?term=Diamond%20K%5BAuthor%5D&cauthor=true&cauthor_uid=21435728) et al. Sleep disturbance relates to neuropsychological functioning in late-life depression. J Affect Disord 2011;132:139-45.
10. Seelye A, Mattek N, Howieson D, Riley T, Wild K, Kaye J. The impact of sleep on neuropsychological performance in cognitively intact older adults using a novel in-home sensor-based sleep assessment approach. Clin Neuropsychol 2015;29:1-14[Shin HY](http://www.ncbi.nlm.nih.gov/pubmed/?term=Shin%20HY%5BAuthor%5D&cauthor=true&cauthor_uid=25045372), [Han HJ](http://www.ncbi.nlm.nih.gov/pubmed/?term=Han%20HJ%5BAuthor%5D&cauthor=true&cauthor_uid=25045372), [Shin DJ](http://www.ncbi.nlm.nih.gov/pubmed/?term=Shin%20DJ%5BAuthor%5D&cauthor=true&cauthor_uid=25045372), [Park HM](http://www.ncbi.nlm.nih.gov/pubmed/?term=Park%20HM%5BAuthor%5D&cauthor=true&cauthor_uid=25045372), [Lee YB](http://www.ncbi.nlm.nih.gov/pubmed/?term=Lee%20YB%5BAuthor%5D&cauthor=true&cauthor_uid=25045372), [Park KH](http://www.ncbi.nlm.nih.gov/pubmed/?term=Park%20KH%5BAuthor%5D&cauthor=true&cauthor_uid=25045372). Sleep problems associated with behavioral and psychological symptoms as well as cognitive functions in Alzheimer’s disease. J Clin Neurol 2014;10:203-209.
11. [Scullin MK](http://www.ncbi.nlm.nih.gov/pubmed/?term=Scullin%20MK%5BAuthor%5D&cauthor=true&cauthor_uid=25588355), [Fairley JA](http://www.ncbi.nlm.nih.gov/pubmed/?term=Fairley%20JA%5BAuthor%5D&cauthor=true&cauthor_uid=25588355), [Trotti LM](http://www.ncbi.nlm.nih.gov/pubmed/?term=Trotti%20LM%5BAuthor%5D&cauthor=true&cauthor_uid=25588355), [Goldstein FC](http://www.ncbi.nlm.nih.gov/pubmed/?term=Goldstein%20FC%5BAuthor%5D&cauthor=true&cauthor_uid=25588355), [Factor SA](http://www.ncbi.nlm.nih.gov/pubmed/?term=Factor%20SA%5BAuthor%5D&cauthor=true&cauthor_uid=25588355), [Bliwise DL](http://www.ncbi.nlm.nih.gov/pubmed/?term=Bliwise%20DL%5BAuthor%5D&cauthor=true&cauthor_uid=25588355). Sleep correlates of trait executive function and memory in Parkinson’s disease. J Parkinsons Dis 2015;5:49-54.
12. [Stavitsky K](http://www.ncbi.nlm.nih.gov/pubmed/?term=Stavitsky%20K%5BAuthor%5D&cauthor=true&cauthor_uid=22152279), [Neargarder S](http://www.ncbi.nlm.nih.gov/pubmed/?term=Neargarder%20S%5BAuthor%5D&cauthor=true&cauthor_uid=22152279), [Bogdanova Y](http://www.ncbi.nlm.nih.gov/pubmed/?term=Bogdanova%20Y%5BAuthor%5D&cauthor=true&cauthor_uid=22152279), [McNamara P](http://www.ncbi.nlm.nih.gov/pubmed/?term=McNamara%20P%5BAuthor%5D&cauthor=true&cauthor_uid=22152279), [Cronin-Golomb A](http://www.ncbi.nlm.nih.gov/pubmed/?term=Cronin-Golomb%20A%5BAuthor%5D&cauthor=true&cauthor_uid=22152279). The impact of sleep quality on cognitive functioning in Parkinson’s disease. J Int Neuropsychol Soc 2012;18:108-117.
13. [Naismith SL](http://www.ncbi.nlm.nih.gov/pubmed/?term=Naismith%20SL%5BAuthor%5D&cauthor=true&cauthor_uid=19128840), [Norrie L](http://www.ncbi.nlm.nih.gov/pubmed/?term=Norrie%20L%5BAuthor%5D&cauthor=true&cauthor_uid=19128840), [Lewis SJ](http://www.ncbi.nlm.nih.gov/pubmed/?term=Lewis%20SJ%5BAuthor%5D&cauthor=true&cauthor_uid=19128840), [Rogers NL](http://www.ncbi.nlm.nih.gov/pubmed/?term=Rogers%20NL%5BAuthor%5D&cauthor=true&cauthor_uid=19128840), [Scott EM](http://www.ncbi.nlm.nih.gov/pubmed/?term=Scott%20EM%5BAuthor%5D&cauthor=true&cauthor_uid=19128840), [Hickie IB](http://www.ncbi.nlm.nih.gov/pubmed/?term=Hickie%20IB%5BAuthor%5D&cauthor=true&cauthor_uid=19128840). Does sleep disturbance mediate neuropsychological functioning in older people with depression? J Affect Disord 2009;116:139-43.
14. Biddle DJ, Naismith SL, Griffiths KM, Christensen H, Hickie IB, Glozier NS. Associations of objective and subjective sleep disturbance with cognitive function in older men with comorbid depression and insomnia. Sleep Health 2017;3:178-183.
15. Suh S, Kim H, Dang-Vu TT, Joo E, Shin C. Cortical Thinning and Altered Cortico-Cortical Structural Covariance of the Default Mode Network in Patients with Persistent Insomnia Symptoms. Sleep 2016;39:161-171.

*Not including a control group*

1. [Chaudhary](http://www.ncbi.nlm.nih.gov/pubmed/?term=Chaudhary%20NS%5Bauth%5D) NS, [Chakravorty](http://www.ncbi.nlm.nih.gov/pubmed/?term=Chakravorty%20S%5Bauth%5D) S, [Evenden](http://www.ncbi.nlm.nih.gov/pubmed/?term=Evenden%20JL%5Bauth%5D) JL, [Sanuck](http://www.ncbi.nlm.nih.gov/pubmed/?term=Sanuck%20N%5Bauth%5D) N. Insomnia severity is associated with decreased executive functioning in patients with suicidal ideation and drug abuse. Prim Care Companion CNS Disord 2013;15:PCC.13l01548.
2. Hart RP, Morin CM, BestAM. Neuropsychological performance in elderly insomnia patients. Aging Neuropsychol Cogn 1995;2:268-78.
3. [Tek C](http://www.ncbi.nlm.nih.gov/pubmed/?term=Tek%20C%5BAuthor%5D&cauthor=true&cauthor_uid=25454802), [Palmese LB](http://www.ncbi.nlm.nih.gov/pubmed/?term=Palmese%20LB%5BAuthor%5D&cauthor=true&cauthor_uid=25454802), [Krystal AD](http://www.ncbi.nlm.nih.gov/pubmed/?term=Krystal%20AD%5BAuthor%5D&cauthor=true&cauthor_uid=25454802), [Srihari VH](http://www.ncbi.nlm.nih.gov/pubmed/?term=Srihari%20VH%5BAuthor%5D&cauthor=true&cauthor_uid=25454802), [DeGeorge PC](http://www.ncbi.nlm.nih.gov/pubmed/?term=DeGeorge%20PC%5BAuthor%5D&cauthor=true&cauthor_uid=25454802), [Reutenauer EL](http://www.ncbi.nlm.nih.gov/pubmed/?term=Reutenauer%20EL%5BAuthor%5D&cauthor=true&cauthor_uid=25454802). The impact of eszopiclone on sleep and cognition in patients with schizophrenia and insomnia: a double-blind, randomised, placebo-controlled trial. Schizophr Res 2014;160:180-5.
4. Ling, A, Lim ML, Gwee X, Ho RCM, Collinson SL, Ng TP. Insomnia and daytime neuropsychological test performance in older adults. Sleep Med 2016;17:7-12.
5. Bernstein JPK, Calamia M, Keller JN. Multiple self-reported sleep measures are differentially associated with cognitive performance in community-dwelling nondemented elderly. Neuropsychol 2018;32:220-229.

*Same sample of an included study*

1. [Bastien CH](http://www.ncbi.nlm.nih.gov/pubmed/?term=Bastien%20CH%5BAuthor%5D&cauthor=true&cauthor_uid=12505554), [Fortier-Brochu E](http://www.ncbi.nlm.nih.gov/pubmed/?term=Fortier-Brochu%20E%5BAuthor%5D&cauthor=true&cauthor_uid=12505554), [Rioux I](http://www.ncbi.nlm.nih.gov/pubmed/?term=Rioux%20I%5BAuthor%5D&cauthor=true&cauthor_uid=12505554), [LeBlanc M](http://www.ncbi.nlm.nih.gov/pubmed/?term=LeBlanc%20M%5BAuthor%5D&cauthor=true&cauthor_uid=12505554), [Daley M](http://www.ncbi.nlm.nih.gov/pubmed/?term=Daley%20M%5BAuthor%5D&cauthor=true&cauthor_uid=12505554), [Morin CM](http://www.ncbi.nlm.nih.gov/pubmed/?term=Morin%20CM%5BAuthor%5D&cauthor=true&cauthor_uid=12505554). Cognitive performance and sleep quality in the elderly suffering from chronic insomnia: relationship between objective and subjective measures. J Psychosom Res 2003;54:39-49. Same population of Vigola et al. 2000.

*Allowing medication intake or psychoactive substance consumptions (alchol, caffeine)*

1. Boyle J, Trick L, Johnsen S, Roach J, Rubens R. Next-day cognition, psychomotor function, and driving-related skills following nighttime administration of eszopiclone. Hum Psychoparmacol Clin Exp 2008;23:385-97.
2. Caplette-Gingras A, Savard J, Savard MH, Ivers H. Is insomnia associated with cognitive impairments breast cancer patients? Behav Sleep Med 2012;11:239-57.
3. Drummond SPA, Walker M, Almklov E, Campos M, Anderson DE, Straus LD. Neural correlates of working memory performance in primary insomnia. Sleep 2013;36:1307-16.
4. Goldman-Mellor S, Caspi A, Gregory AM, Harrington HL, Poulton R, Moffitt TE. Is insomnia associated with deficits in neuropsychological functioning? Evidence from a population-based study. Sleep 2015;38:623-31.
5. Leufkens TRM, Ramaekers JG, de Weerd AW, Riedel WJ, Vermeeren A. On-the-road driving performance and driving-related skills in older untreated insomnia patients and chronic users of hypnotics. Psychopharmacology 2014;231:2851-65.
6. Orff HJ, Drummond SPA, Nowakowski S, Perlis ML. Discrepancy between subjective symptomatology and objective neurpsychological performance in insomnia. Sleep 2007;30:1205-11.
7. Varkevisser M, Kerkhof GA. Chronic insomnia and performance in a 24-h constant routine study. J Sleep Res 2005;14:49-59.
8. Zimmerman ME, Bigal ME, Katz MJ, Brickman AM, Lipton RB. J Int Neuropsychol Soc 2012;18:461-70.

**3. PRISMA Checklist (2009).**

| **Section/topic** | **#** | **Checklist item** | **Reported on page #** |
| --- | --- | --- | --- |
| **TITLE** | | |  |
| Title | 1 | Identify the report as a systematic review, meta-analysis, or both. | 1 |
| **ABSTRACT** | | |  |
| Structured summary | 2 | Provide a structured summary including, as applicable: background; objectives; data sources; study eligibility criteria, participants, and interventions; study appraisal and synthesis methods; results; limitations; conclusions and implications of key findings; systematic review registration number. | 2 |
| **INTRODUCTION** | | |  |
| Rationale | 3 | Describe the rationale for the review in the context of what is already known. | 3-6 |
| Objectives | 4 | Provide an explicit statement of questions being addressed with reference to participants, interventions, comparisons, outcomes, and study design (PICOS). | 6 |
| **METHODS** | | |  |
| Protocol and registration | 5 | Indicate if a review protocol exists, if and where it can be accessed (e.g., Web address), and, if available, provide registration information including registration number. | / |
| Eligibility criteria | 6 | Specify study characteristics (e.g., PICOS, length of follow-up) and report characteristics (e.g., years considered, language, publication status) used as criteria for eligibility, giving rationale. | 8 |
| Information sources | 7 | Describe all information sources (e.g., databases with dates of coverage, contact with study authors to identify additional studies) in the search and date last searched. | 7-8 |
| Search | 8 | Present full electronic search strategy for at least one database, including any limits used, such that it could be repeated. | 7-8 |
| Study selection | 9 | State the process for selecting studies (i.e., screening, eligibility, included in systematic review, and, if applicable, included in the meta-analysis). | 7-8 |
| Data collection process | 10 | Describe method of data extraction from reports (e.g., piloted forms, independently, in duplicate) and any processes for obtaining and confirming data from investigators. | 8-9 |
| Data items | 11 | List and define all variables for which data were sought (e.g., PICOS, funding sources) and any assumptions and simplifications made. | 8-9 |
| Risk of bias in individual studies | 12 | Describe methods used for assessing risk of bias of individual studies (including specification of whether this was done at the study or outcome level), and how this information is to be used in any data synthesis. | 9 |
| Summary measures | 13 | State the principal summary measures (e.g., risk ratio, difference in means). | 9-10 |
| Synthesis of results | 14 | Describe the methods of handling data and combining results of studies, if done, including measures of consistency (e.g., I^2^) for each meta-analysis. | 9-10 |
| Section/topic | # | Checklist item | Reported on page # |
| Risk of bias across studies | 15 | Specify any assessment of risk of bias that may affect the cumulative evidence (e.g., publication bias, selective reporting within studies). | 9-10 |
| Additional analyses | 16 | Describe methods of additional analyses (e.g., sensitivity or subgroup analyses, meta-regression), if done, indicating which were pre-specified. | 9-10 |
| **RESULTS** | | |  |
| Study selection | 17 | Give numbers of studies screened, assessed for eligibility, and included in the review, with reasons for exclusions at each stage, ideally with a flow diagram. | 11 |
| Study characteristics | 18 | For each study, present characteristics for which data were extracted (e.g., study size, PICOS, follow-up period) and provide the citations. | 11 |
| Risk of bias within studies | 19 | Present data on risk of bias of each study and, if available, any outcome level assessment (see item 12). | 11 |
| Results of individual studies | 20 | For all outcomes considered (benefits or harms), present, for each study: (a) simple summary data for each intervention group (b) effect estimates and confidence intervals, ideally with a forest plot. | 11-19 |
| Synthesis of results | 21 | Present results of each meta-analysis done, including confidence intervals and measures of consistency. | 16-19 |
| Risk of bias across studies | 22 | Present results of any assessment of risk of bias across studies (see Item 15). | S1 |
| Additional analysis | 23 | Give results of additional analyses, if done (e.g., sensitivity or subgroup analyses, meta-regression [see Item 16]). | 16-19 |
| **DISCUSSION** | | |  |
| Summary of evidence | 24 | Summarize the main findings including the strength of evidence for each main outcome; consider their relevance to key groups (e.g., healthcare providers, users, and policy makers). | 19-21 |
| Limitations | 25 | Discuss limitations at study and outcome level (e.g., risk of bias), and at review-level (e.g., incomplete retrieval of identified research, reporting bias). | 21 |
| Conclusions | 26 | Provide a general interpretation of the results in the context of other evidence, and implications for future research. | 22 |
| **FUNDING** | | |  |
| Funding | 27 | Describe sources of funding for the systematic review and other support (e.g., supply of data); role of funders for the systematic review. | / |

*From:*  Moher D, Liberati A, Tetzlaff J, Altman DG, The PRISMA Group (2009). Preferred Reporting Items for Systematic Reviews and Meta-Analyses: The PRISMA Statement. PLoS Med 6(7): e1000097. doi:10.1371/journal.pmed1000097

For more information, visit: **www.prisma-statement.org**.

Abbreviations: Std diff in means= standardised difference in mean.
